# ASAS-NANP symposium: mathematical modeling in animal nutrition: agent‑based modeling of nutrient requirements and growth performance in growing–finishing pigs for sustainable production systems

**DOI:** 10.1093/jas/skaf443

**Published:** 2025-12-18

**Authors:** Atefeh Rahimifar, Karun Kaniyamattam, Jeffrey G Wiegert, Luis O Tedeschi

**Affiliations:** Department of Electrical Engineering, Karoon Institute of Higher Education, Ahvaz; Department of Animal Science, Texas A&M University, College Station, TX 77843-2471; Department of Animal Science, Texas A&M University, College Station, TX 77843-2471; Department of Animal Science, Texas A&M University, College Station, TX 77843-2471

**Keywords:** agent-based modeling, growing-finishing pigs, pork production management, nutrition requirement, swine nutrition

## Abstract

The nutritional requirements of growing-finishing pigs are based on a complex interplay of parameters like feed intake (FI), metabolism, and other environmental conditions. We developed an individual-based model to estimate the growth performance and nutritional requirements of growing-finishing pigs. Our model implementation follows the principles and equations published by the 2012 Swine National Research Council (NRC) model, focusing on reflecting the attributes of each pig and its interaction with the environment. The proposed swine nutrition system (SNS) utilized an agent-based model framework and was developed using NetLogo to simulate the dynamics of pig nutrition systems. The main factors incorporated in the model were body weight (BW) gain (BWG), start and finish BW, and the sex of the pigs. Results showed that the model can accurately estimate foundational parameters of pigs’ biological growth, including BW, FI, metabolizable energy intake (MEI), protein deposition (Pd), and lipid deposition (Ld). In addition, the daily requirements, such as amino acid, calcium, and phosphorus requirements, were calculated separately for each pig. The model accurately demonstrates known differences in pig growth characteristics, such as greater Ld in barrows, greater daily protein accretion, and dietary standardized ileal digestible lysine requirements in boars. The proposed model was evaluated by comparing SNS predictions’ correlation and coefficient of determination (*r*^2^) to those of the 2012 Swine NRC model’s predictions. The simulations were conducted for 500 pigs to demonstrate the repeatability of the SNS. The average, standard deviation, and the 95% confidence interval were obtained over the growth period of the pigs for the parameters, such as BWG, BW, Pd, Ld, MEI, and FI. The correlation between BWG from the SNS and BWG from the NRC was *r*^2^ = 0.84 for gilts, *r*^2^ = 0.93 for barrows, and *r*^2^ = 0.93 for boars. Also, the direct comparison between the SNS and the 2012 Swine NRC indicated an *r*^2^ > 0.99 for the three sexes. SNS enabled the simulation of individual animal behavior, nutrient partitioning, and variability in growth performance, which are capabilities not afforded by traditional aggregate models. Thus, real-world implementation of SNS might improve feeding and management strategies within commercial swine production systems, leading to greater production efficiency and sustainability in the pork sector.

## Teaser

Agent-based modeling is an innovative modeling technique that aids in sustainable pig farming, by precisely accounting for individual animal nutrient needs, significantly reducing resource inefficiencies, and improving production efficiency. This technique is crucial for more sustainable and economically viable pork production.

## Introduction

The United States is ranked third among pork producers worldwide and second among pork exporters, accounting for approximately 11% of global pork production and 31% of pork exports (Conrad et al., 2024). Due to population growth and nutrition pattern changes in many countries, global pork demand has risen by 140% since the 1960s (Kim et al., 2024), which compels the swine industry to rapidly adopt sustainable feeding practices to ensure long-term viability amid resource constraints. In this context, the path toward sustainability depends on three issues: economic viability of operations, minimal environmental impacts, and socially responsible farming practices (Kruger et al., 2022; Vonderohe et al., 2022). Literature indicates that between 65% and 80% of swine production expenses are associated with nutrition (Alves et al., 2022), out of which the feeding expenses during the growing-finishing phase are 75% of total feeding expenses (Yang et al., 2023). Appropriate nutrition prevents growth-limiting deficiencies and optimizes muscle development, enabling pigs to reach market weight efficiently (Solà-Oriol and Gasa, 2017). This is especially important for evolving genotypes, which tend to grow faster than those in older models (Schinckel et al., 2012; Wang et al., 2022). However, excesses primarily cause economic and environmental waste without necessarily increasing growth rates beyond supported genetic potential (Portejoie et al., 2004; Andretta et al., 2016; Remus et al., 2020). Therefore, precise feeding strategies are crucial for the sustainability of the swine industry, requiring adaptable energy requirement frameworks tailored to genetic differences advancement.

Our group had previously defined the structure of the swine nutrition system (SNS) model as a multi-dimensional and intricate system characterized by some defining features of feed input, nutrient assimilation by swine, waste output, and environmental impacts due for consideration (Rahimifar et al., 2024). The nutritional requirements of pigs vary between animals depending on their age, sex, body weight (BW), production stage (e.g. gestation, lactation, growing-finishing), and environmental factors (Carroll and Allee, 2009). The factors influencing pigs’ dietary requirements include physiological state, potential performance, and environmental conditions. Formulating a suitable ration for pigs is challenging as it requires a deep understanding of nutrient requirements, feed ingredients, and their interactions within the digestive system (Chiba, 2013; Gaillard et al., 2020). Typically, diets are formulated to the average of a population’s nutritional requirements, failing to account for the random natural variation (i.e. the stochastic elements) inherent between entities in biological systems, leading to unacceptable errors that must be addressed. One possible solution is to represent the individual entities and their interaction with each other and the environment to capture the dynamics of the SNS model.

Agent-based modeling (ABM) is a powerful modeling tool that represents the stochasticity of swine systems (Kaniyamattam and Tedeschi, 2023; Tedeschi, 2023; Cercena, 2024). Through the representation of individual pigs as agents in the model, researchers can simulate interactions of pigs, feed intake (FI), nutrient metabolism, and environmental conditions to understand better how those variables affect swine nutrition (Boumans et al., 2018). ABM is suited to capture complexities in the dynamics of the SNS model, given that it represents individual agents and their interactions occurring in a more expansive system (Berger, 2001; Bonabeau, 2002; Grimm and Railsback, 2013). Furthermore, it provides a good description of pig growth patterns that helps to develop accurate feeding programs in a sustainable production system (Sargent, 2010; Pham et al., 2021). This approach allows for the simulation of different scenarios (Jablonski et al., 2018) and provides insights into optimizing feed formulations, feeding strategies, and management practices to improve pig health, performance, and sustainability (Bayram et al., 2023; Akintan et al., 2024; Bayram et al., 2024).

Although all four phases, starter, growing-finishing, gestating, and lactating, are important in pork production, the growing-finishing phase is often considered particularly crucial, as it is the longest and the most expensive phase (Gentry et al., 2008), and it directly influences the efficiency of pork production, the quality of the final product, and the operation’s profitability. Efficient nutrition and effective management in the growing-finishing phase are essential for maximizing growth performance and minimizing pork production expenses (Tokach et al., 2012). Furthermore, to produce high-quality pork, it is necessary to investigate the effect of nutrition, management, and environmental conditions on body composition and meat quality (Monteiro et al., 2017).

This article describes an ABM-based approach to studying and managing growing-finishing pig production systems. We created an ABM-based SNS modeled after the NRC (2012) Swine Nutrition Model to perform *in silico* experimentation to investigate the following objectives: a) investigate the effect of the individual parameters of pigs such as age, sex, BW, and metabolism on growth performance, b) make a precise assessment of nutrient requirements of individual pigs, c) create a cost-effective platform for testing different feeding strategies for pig production, and d) move toward an optimal sustainable SNS.

## Materials and Methods

### ABM-based SNS model development

To realistically replicate the nutrient requirements and growth performance of growing-finishing pigs, we developed an ABM-based SNS on the principles and equations from the nutrient requirements of swine published by the National Research Council (NRC, 2012). Our SNS model is a simulation environment that captures the complex interactions between pigs’ nutrient intake, metabolism, and growth performance. In this simulation, each pig is represented as an autonomous agent with unique attributes and behaviors that affect its growth and development over time (Wilensky, 2015; Railsback and Grimm, 2019; Tedeschi, 2023). This micro-level representation allowed for a more sensible and comprehensive simulation of the underlying biological processes and the evaluation of potential ­management strategies. The integration of the NRC recommendations in the SNS simulations was achieved through the following five steps, as discussed below.

#### Identification of relevant NRC equations and relationships

We identified the key equations, parameters, and relationships that govern pigs’ growth, body composition, energy requirements, and nutrient partitioning based on the NRC (2012). This included equations for calculating critical body parameters, for instance, protein deposition (Pd), lipid deposition (Ld), ash, and water content, as well as the environmental factors’ impact, such as temperature, on the pigs’ energy requirements and FI.

#### Translation of NRC concepts into ABM constructs

Then, the NRC (2012) policies and equations were rephrased into ABM, which was carried out using NetLogo (Wilensky, 1999), an ABM platform known for its versatility and capability to simulate dynamic and complex systems. The individual pig agents, their attributes, and their dynamic behaviors were defined over time. Thus, the integrated SNS was designed to simulate the growth and development of each pig agent.

#### Sex-specific characteristics

The simulation model incorporated three sexes of growing-finishing pigs: gilt, barrow, and boar. Each sex was represented by a different agent type, enabling the simulation of their unique growth and production characteristics and potential sex-specific responses to various feeding and management practices. The model captured the sex-specific differences in body composition and energy and nutrient requirements.

#### Spatial structure and environmental factors

Depending on the user’s intention, the simulated environment was divided into discrete patches, each with attributes that could represent a distinct farm, barn, or pen. This attribute helps to monitor the influence of environmental conditions and resource availability on the growth and production of pigs. This spatial structure allowed for incorporating factors like temperature and housing, which can affect the pigs’ energy requirements and FI, as well as potential constraints or limitations on resources and space.

#### Comprehensive attribute representation

Each pig was represented with a comprehensive set of attributes, including BW, body composition (e.g. whole-body protein mass (BP), whole-body lipid mass (BL), whole-body ash mass (BA), whole-body water mass (BWt)), FI, energy requirements, vitamins, minerals, and various other physiological parameters. The dynamic changes in these attributes over time were simulated, allowing for a detailed representation of the pigs’ growth and production performance individually.

### Model parameters and calibrations

**Figure 1.**
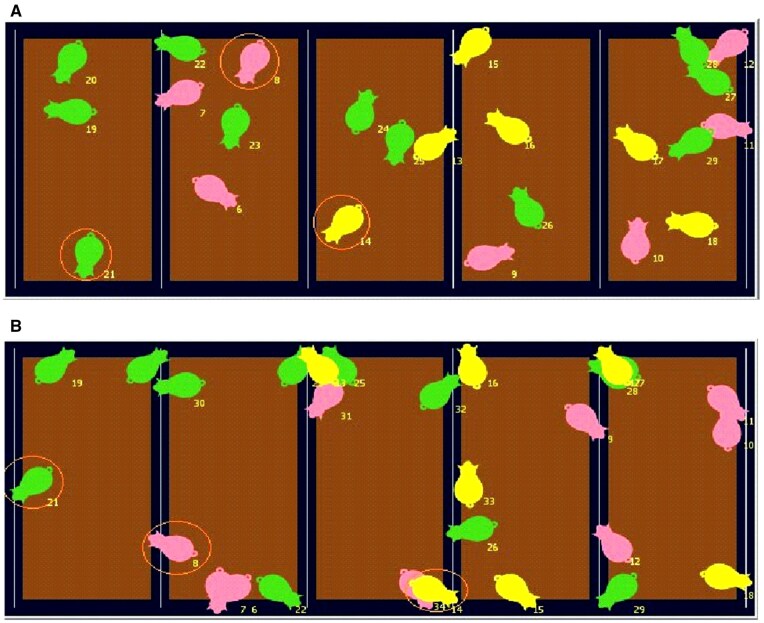
Example simulation interface showing pig movements; pen configurations (e.g. mixed sexes) are user-configurable for research scenarios, where three different sexes are depicted (Gilts, barrows, and boars). (A) The 1st day of simulation. (B) The 82nd day of simulation. Orange circles display individual pig movements through the pens.

Mathematical modeling integrates powerful data-driven tools and knowledge-driven approaches to reach functional models that are sustainable and resilient (Tedeschi, 2019). The SNS consisted of individual growing-finishing pig agents capable of movement, interaction with their environment, feed consumption, nutrient metabolism, and growth based on their physiology and nutritional status. Each pig in the model began with an initial BW of 20 kg and finished with a final BW of 130 kg (Cromwell, 1999). The final BW of 130 kg reflects industry practices, though NRC (2012) models up to 135 kg. Pigs were fed for a certain number of days on the farm until they reached the final BW. The simulated environment was divided into discrete patches, each representing a distinct farm, barn, or pen. This structure allowed the incorporation of environmental factors like temperature and housing, which influence energy requirements and FI. Although the model permits user-defined pen configurations (including mixed-sex groups for research purposes), all simulations in this study utilized sex-segregated housing reflecting commercial standards. Figure 1 illustrates example agent movements and pen setups within the NetLogo interface.

**Figure 2.**
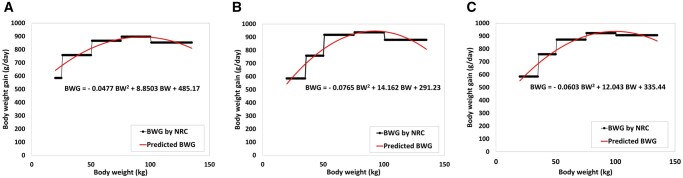
Predicted body weight gain (BWG) was generated using polynomial regressio n. The line extracted equation that was used to calculate BWG in the Swine Nutrition System model simulations. The bars show the phased BWG used in NRC (2012). (A) BWG for gilts, (B) for barrows, (C) for boars

The ME content of the feed was set at 3,300 Kcal/kg, with a 5% allowance for feed wastage. Body weight gain (BWG) is one of the fundamental attributes of pigs in assessing feed efficiency and nutrition systems (Noblet and Van Milgen, 2004; Patience et al., 2015; Tang et al., 2022). For the SNS model, the estimated BWG of growing-finishing pigs was obtained from NRC (2012), considering the BW, growth phase, and sex of the pigs. A polynomial regression was applied to create an equation to illustrate the variation of BWG based on the obtained data for each pig during its life cycle (Fig. 2). To mimic the effect of stochastic factors, a range of probable deviations of BWG for different pigs in each group was considered. Therefore, all pigs will have unique growth conditions even if they have the same sex, age, and are subject to the same production environment. All other body composition parameters were calculated using BWG. Therefore, it can be expected that this random consideration in BWG will affect the results of all growth processes, resulting in individual variations in performance.

Supplementary Appendix S1 includes a simple algorithm that describes the daily processes that occur dynamically on a pig farm, with a special burden on feeding. This outlines the step-by-step process of the simulation, starting with the initialization of the environment and global parameters. Algorithm 1 iterates through a predefined number of time steps, during which it gathers critical information about each pig, assesses their status based on various metrics such as BW, sex, and environmental conditions, and determines their actions within the barn. The algorithm also incorporates mechanisms for updating the pigs’ attributes and requirements, environmental conditions, and removing pigs that reach their target BW. Finally, it records essential outputs and generates reports and visualizations to facilitate analysis of the simulation results.

### Model calculations

#### Body composition calculations

Individual level (*i*) growth performance and daily (*j*) requirements were represented in the equations listed in Table 1. They provide a broad basis for analyzing body composition parameters essential in swine nutrition, focused on growth performance. The SNS model starts with the calculation of BWG using equation (1.1), which quantifies the BW gained over a specific feeding period and considers it a baseline for assessing total growth efficiency. Equation (1.2) uses BWG to get a current BW reflecting the actual daily growth of pigs. In equation (1.3), the empty BW is calculated based on BP, BL, BWt, and BA, which gives information on the physiological status of the pig. Equation (1.4) captures the water content in the body, thus estimating the hydration status of the pig short of water, which is a key parameter for metabolic functions and general health. Furthermore, equation (1.5), through the estimation of the BA, establishes the mineral contents, which are important for determining nutrient utilization and skeletal health. The gut fill (Gutf) value, presented in equation (1.6), provides insights into the volume of content in the gastrointestinal tract (GIT), influencing BW and food conversion efficiency. Probe backfat thickness (PBT) indicates the deposition of fat relative to body length and weight, which is a valuable measure for assessing carcass quality and market readiness. PBT can be estimated using equation (1.7). In addition, Pd and BP, calculated by equations (1.8) and (1.9), respectively, demonstrate the dynamics of muscle build-up, which is critical for evaluating growth efficacy and feed conversion ratios. Finally, Ld and BL obtained by equations (1.10) and (1.11) permit monitoring of fat storage processes, thus leading the diet to be modified to achieve desired BW and body composition outcomes. The proposed model applies these equations to ensure that the most significant components affecting growing-finishing pig body compositions have been accounted for.

**Table 1.** Body composition calculations, adopted to individual pig nutrition dynamics for the Swine Nutrition System model, adopted from the National Research Council (**NRC**, 2012) model

Parameter	Description	Equation^1^	Equation number
***BWG***	Body weight gain	${\mathrm{BWG}}_{ij}(g/\mathrm{day})=485.17+8.8503\times {BW}_{(i-1)j}-0.0477\times {BW}_{(i-1)j}^{2}$	(1.1)
***BW***	Body weight	${BW}_{ij}(kg)={BW}_{(i-1)j}+{\mathrm{BWG}}_{ij}$	(1.2)
***EBW***	Empty body weight	${\mathrm{EBW}}_{ij}\;(kg)={BP}_{ij}+{BL}_{ij}+{\mathrm{BWt}}_{ij}+{BA}_{ij}$	(1.3)
***BWt***	Whole-body water mass	${\mathrm{BWt}\;}_{ij}\;(kg)=(4.322+(0.0044\;\&\times \;{{Pd}_{\max}}_{j}))\;\&\times \;P_{ij}^{0.855}$	(1.4)
***BA***	Whole-body ash mass	${BA\;}_{ij}\;(kg)=0.189\;\&\times \;{BP}_{ij}$	(1.5)
***Gutf***	Gut fill	${\mathrm{Gutf}}_{ij}\;(kg)=0.277\&\times \;{BW}_{0j}^{0.612}$	(1.6)
***PBT***	Probe backfat thickness	${\mathrm{PBT}}_{ij}\;(mm)=-5+\frac{12.3\;\&\times \;{BL}_{ij}}{{BP}_{ij}}+(0.13\;\&\times \;{BP}_{ij})$	(1.7)
***Pd***	Protein deposition^2^ (Depends on sex)	${Pd\;}_{ij}(g/d)=(a)\&\times [b+(c\;\&\times \;{BW}_{ij})+(d\&\times \;{BW}_{ij}^{2})+(e\times {BW}_{ij}^{3})]$	(1.8)
***BP***	Whole-body protein mass	${BP}_{ij}={BP}_{(i-1)j}+{Pd}_{ij}$	(1.9)
***Ld***	Lipid deposition	${Ld}_{ij}\;(g/d)=\frac{\left({\mathrm{MEint}}_{ij}-{\mathrm{maintenME}}_{ij}-{Pd}_{ij}\times 10.6\right)}{12.5}$	(1.10)
***BL***	Whole-body lipid mass	${BL}_{ij}={BL}_{(i-1)j}+{Ld}_{ij}$	(1.11)

1 *i*: The day under feeding; *j*: Pig’s ID.

2 For gilts: *a *= 137, *b *= 0.7066, *c *= 0.013289, *d *= −1.3120 × 10^−4^, *e *= 2.8627 × 10^−7^;

For barrows: *a *= 133, *b *= 0.7078, *c *= 0.013764, *d *= −1.4211 × 10^−4^, *e *= 3.2698 × 10^−7^;

For boars: *a *= 151, *b *= 0.6558, *c *= 0.012740, *d *= −1.0390 × 10^−4^, *e *= 1.64001 × 10^−7^.

#### Energy and FI calculations

The optimization of pork production systems requires knowledge of pig FIs, growth performance, estimated feed measurements, and energetic intake (Schinckel et al., 2012). Energy intake and FI calculations help to know the metabolic needs of pigs, therefore meeting the growth and health requirements. The key equations related to energy intake and FI appropriate to the physiological dynamics of swine nutrition are provided in Table 2. Metabolizable energy intake (MEI), calculated by equation (2.1), illustrates the energy balance and the efficiency of nutrient utilization amongst pigs. Where specific coefficients (*a*, *b*, and *c*) are tailored for varying sexes of pigs, including gilts, barrows, and boars. ME is the animal’s energy for maintenance, growth, and reproduction after the losses in the urine and gaseous digestion products are accounted for. The dependence of the ME calculated by the model on the BW and sex of the animal illustrates the model’s ability to take into account the subtle physiological differences of pigs to examine individual aspects of the herd. Equation (2.2) determines FI, where coefficients (*x*, *y*, and *z*) are tailored for varying sexes of pigs (Schinckel et al., 2009). Equation (2.3) estimated the lower critical temperature (LCT), which addresses the effect of environmental temperature on MEI. In fact, LCT is the critical temperature under the concept of maintaining thermal comfort through pigs’ energy consumption. Equation (2.4) can be used to estimate the influence of pig density on MEI by quantifying the minimum space allocation that leads to maximum MEI. This emphasizes the space factor in livestock husbandry, which affects overall productivity. Equation (2.5) gives maximum daily FI based on BW and environmental conditions, underlining the interlink between metabolic requirements and environmental temperature.

**Table 2.** Energy intake and feed intake calculations were adopted to simulate individual pig nutrition dynamics for the Swine Nutrition System model, adopted from the National Research Council (NRC, 2012) model

Parameter	Description	Equation^1^	Equation number
***MEI***	Metabolizable energy intake^2^	${\mathrm{MEI}}_{ij}\;(\mathrm{kcal}/d)=a\&\times \;\{1-\;\text{exp}\;[-\;\text{exp}\;\left(b\right)\&\times {\;BW}_{ij}^{c}]\}$	(2.1)
***FI***	Feed intake	${FI}_{ij}(kg/d)=x\times (1-\;\text{exp}\;\left(-\;\text{exp}\;\left(y\right)\times {BW}^{z}\right))$	(2.2)
***LCT***	Lower critical temperature^3^	${\mathrm{LCT}\;}_{ij}(˚C)=17.9-0.0375\;\&\times \;{BW}_{ij}$	(2.3)
***Minspace***	Minimum space for maximum MEI	${\mathrm{Minspace}}_{ij}\;(m^{2}/\mathrm{pig})=0.0336\&\times \;{BW}_{ij}^{0.667}$	(2.4)
***MaxFI***	Maximum daily feed intake	${\mathrm{MaxFI}}_{ij}\;(g/d)=(111\&\times \;{BW}_{ij}^{0.803})[1+({\mathrm{LCT}}_{ij}-T)\&\times \;0.025]$	(2.5)
***StdmME***	Standard maintenance ME requirements	${\mathrm{StdmME}}_{ij}\;(\mathrm{kcal}/\mathrm{day})=197\&\times \;{BW}_{ij}^{0.60}$	(2.6)
***MEtherm***	ME requirements for thermogenesis	${\mathrm{MEtherm}\;}_{ij}\;(\mathrm{kcal}/d)=0.07425\&\times \;({\mathrm{LCT}}_{ij}-T)\&\times {\;\mathrm{StdmME}}_{ij}$	(2.7)
***mME***	Maintenance ME requirements^4^	${\mathrm{mME}\;}_{ij}\;(\mathrm{kcal}/d)={\mathrm{tdmME}}_{ij}+{\mathrm{MEtherm}\;}_{ij}+\mathrm{adj}$	(2.8)

1 *i*: The day under feeding; *j*: Pig’s ID.

2 Gilts: *a *= 10,967, *b* = −3.803, *c *= 0.9072, x = 2.755, y = −4.755, z = 1.214; Barrows: *a *= 10447, *b* =—4.283, *c *= 1.0843, x = 2.88, y = -5.921, z = 1.512; Boars: *a *= 10,638, b = −3.803, *c *= 0.9072.

3 *T*: Environmental temperature, °C.

4 *adj*: ME requirements for increased activity or genotype adjustment.

Equation (2.6) gives the standard maintenance ME requirements of pigs as a function of BW. Equation (2.7) gives ME requirements that consider thermogenesis, which is a function of LCT, environmental temperature, and standard maintenance ME requirements. This reflects that the animal’s body consumes significant energy to maintain body temperature in an unfamiliar environment. Lastly, maintenance ME requirements were calculated using the standard maintenance and thermogenic factors by considering adjustments of activity and genotype via equation (2.8) to give a relatively complete assessment of energy intake for any individual pig in a given situation.

#### Body requirements calculations

Table 3 contains the equations for calculating the growing-finishing pigs’ lysine, phosphorus, and calcium requirements. Equation (3.1) refers to the GIT lysine losses, incorporating a constant factor alongside the feed dry matter (FDM) and FI. This bears out the connectedness of these variables with the losses of nutrients that can happen within the GIT. Following this, equation (3.2) is assigned to integument lysine losses as a function of BW, signifying the effects of metabolic scaling pertinent to lysine loss concerning body mass. Equation (3.3) obtains the summation of standardized ileal digestible (SID) lysine requirements for both GIT and integument losses. Similarly, equation (3.4) computes the SID lysine requirements particularly dependent on Pd, highlighting Pd’s critical role in effective nutrition modeling. Equation (3.5), finally, estimates total SID lysine requirements using equations (3.3) and (3.4). Equation (3.6) determines body phosphorus mass as a quadratic function of BP, which provides practical insights into metabolic processes related to phosphorus production. The FDM intake (FDMI), calculated by multiplying FDM by FI, as illustrated by equation (3.7), indicates the amount of dry feed consumed by pigs. This has a direct effect on growth, feed conversion, and health. Then, equation (3.8) details standardized total tract digestible (STTD) P requirements in maximum P retention, FI, and BW, emphasizing how crucial it is to manage phosphorus effectively in swine nutrition. Maximum P retention refers to the highest level of phosphorus an animal can effectively absorb and utilize from its diet while minimizing losses through excretion in urine and feces. Total calcium requirements as a function of STTD P are given by equation (3.9), which is how phosphorus directly influences calcium needs and how these two nutrients correlate with each other. It is essential to emphasize that the formulation of the minerals and vitamins in the nutrition of growing-finishing pigs helps ensure they receive adequate amounts of essential nutrients needed to promote optimal growth, health, and productivity. Meanwhile, equations (3.10) and (3.11) show how BW influenced these dietary requirements for vitamins and minerals. The coefficients p and q for minerals and m and n for vitamins have been expressed in Supplementary Table S1.

**Table 3.** Standardized ileal digestible (SID) Lysin, phosphorus, calcium, mineral, and vitamin equations adopted to simulate individual pig nutrition dynamics for the Swine Nutrition System model, adopted from the National Research Council (**NRC**, 2012) model

Parameter	Description	Equation^1^	Equation number
***GITLys***	Basal endogenous GIT lysine losses	${\mathrm{GITLys}}_{ij}=1.1\times 4.17\times 10^{-4}\times \mathrm{FDM}\times {FI}_{ij}$	(3.1)
***ILys***	Integument lysine losses	${\mathrm{ILys}}_{ij}=0.0045\&\times \;{BW}_{ij}^{0.75}$	(3.2)
***SIDLys1***	SID lysine requirements for GIT plus integument losses	${\mathrm{SIDLys}1}_{ij}=\frac{{\mathrm{GITLys}}_{ij}+{\mathrm{ILys}}_{ij}}{0.75+0.002\;\&\times \;({\mathrm{maxPd}}_{ij}-147.7)}$	(3.3)
***SIDLys2***	SID lysine requirements for Pd	${\mathrm{SIDLys}2}_{ij}=\frac{(0.071\times {Pd}_{ij})\times (1.0547+0.002215\times {BW}_{ij})\;}{0.75+0.002\times ({\mathrm{maxPd}}_{ij}-147.7)}$	(3.4)
***SIDLys***	Total SID lysine requirements	${\mathrm{SIDLys}}_{ij}={\mathrm{SIDLys}1}_{ij}+{\mathrm{SIDLys}2}_{ij}$	(3.5)
***P***	Whole-body phosphorus mass	$P_{ij}=1.1613+26.012\times {BP\;}_{ij}+(0.2299\times {BP\;}_{ij}^{2})$	(3.6)
***FDMI***	Feed dry matter intake	${\mathrm{FDMI}}_{ij}=\mathrm{FDM}\times {FI}_{ij}$	(3.7)
***STTDP***	STTD P requirements	${\mathrm{STTDP}\;}_{ij}=0.85\times [\frac{\left(\max\;P\;\mathrm{retention}\right)}{0.77}+(0.19\times {\mathrm{FDMI}}_{ij})+(0.007\times {BW}_{ij})]$	(3.8)
***Ca***	Total Ca requirements	${Ca}_{ij}=2.15\times {\mathrm{STTDP}}_{ij}$	(3.9)
***Mineral***	Mineral requirements^2, p,^ ^q^	${\mathrm{Mineral}}_{\mathrm{ijk}}=p+q\times ln({BW}_{ij})$	(3.10)
***Vitamin***	Vitamin requirements^2, m, n^	${\mathrm{Vitamin}}_{\mathrm{ijk}}=m+n\times ln({BW}_{ij})$	(3.11)

1 *i*: The day under feeding; *j*: Pig’s ID.

2 *k*: Type of mineral or vitamin.

3 *p, q:* Minerals’ constant coefficients.

4 *m, n*: Vitamins’ constant coefficients.

### Simulation procedure and execution

Supplementary Appendix S2 is an algorithm that demonstrates the feeding procedures and their basic calculations for individual pigs. This pseudocode systematically describes the feeding procedure for all gilts, barrows, and boars within the SNS model. The specific feeding procedures also include calculations about BWG, BW updates for each pig, and body composition assessment, as represented by parameters in Table 1. Energy and nutrient intakes are monitored according to Table 2. In addition, the SNS model calculates the nutritional requirements of swine, including amino acid, calcium, phosphorus, mineral, and vitamin requirements for maintenance and growth.

The model enables user customization of key parameters, including sex distribution, initial and final BWs (default: 20–130 kg), feeding schedules, and environmental conditions to facilitate scenario testing. Dynamic simulation then allowed for the evaluation of the impact of different feeding strategies, management practices, and environmental factors on the growth and production performance of the simulated pigs. The simulation key parameters are presented in Table 4.

**Table 4.** Herd level model parameters, applicable to all growing-finishing pigs, user-adjustable by the swine nutrition system model user in its graphical user interface

Parameter	Amount	Optional
**Number of barns**	5	No
**Number of sexes**	3 (gilt, barrow, boar)	No
**Run time**	140 d	No
**Initial BW**	20 kg	No
**Final BW**	130 kg	No
**ME content**	3300 Kcal	No
**% Feed wastage**	5%	Yes
**% Diet fermentable fiber in feed content**	10.5%	No
**% FDM**	88%	No
**Environmental temperature**	20 ˚C	Yes
**Ractopamine level^1^**	20 mg/kg	Yes
**Starting BW for feeding ractopamine^2^**	78 kg	Yes

1 default = 0 mg/kg; adjustable for historical/global contexts.

2 While feeding ractopamine is banned in US production since 2023, this parameter is retained for simulating foreign production markets, historical data, or research scenarios.

### Model verification

The basis of any successful computational model lies in its ability to reflect the real-world phenomenon it seeks to represent accurately (Tedeschi, 2006). We formally compared the SNS model predictions with the NRC (2012). Because the NRC (2012) does not provide a specific equation for calculating BWG on a daily basis, we developed separate equations for estimating BWG throughout the pigs’ lifespan. We used the estimated BWG values from (NRC, 2012), under ad libitum feeding, to create distinct equations for individual pigs customized for their sex and weight gain phase. The SNS growth model was constructed by selecting the best-fitting polynomial regression equations from the NRC (2012) data (equation (1.1)), as illustrated in Fig. 2. To incorporate stochasticity in BWG in the SNS model, we modeled a daily variation around the BWG equations. The model considers the dynamics of swine systems by generating random BWG based on the sex, BW, and dynamic parameters of animals and establishing a random normal distribution around the BWG equation. Moreover, the initial BW of the pigs follows a normal distribution around the mean of 20 kg. To capture the inherent variability arising from individual-level interactions and stochastic processes within the model, the simulation was repeated 500 times. All attributes of the pigs, including body composition and requirements, were recorded for 130 d. During the simulations, pigs’ BW increased daily based on a random distribution around their specific BWG distribution curve. As all other attributes of the pig are directly dependent on BW, we expect the SNS to extend this stochasticity to all different body composition variables. We statistically evaluated and examined the model’s consistency for the key growth parameters by calculating the average, standard deviation, and 95% confidence interval to assess the SNS’s stochasticity, thereby illustrating the model’s capability to simulate the dynamics of swine systems.

## Results and Discussion

The SNS model successfully simulated growing pigs’ nutrient requirements and growth performance throughout their production cycle. This model was able to depict the complex interactions between pigs, FI, metabolism, and environmental factors and provide an accurate and dynamic representation of the growth and development of pigs. By focusing primarily on known differences in pig growth between sexes, our evaluation highlighted key parameters of pigs in the production cycle and highlighted the ability of the model to show the individual behaviors of pigs.

**Figure 3.**
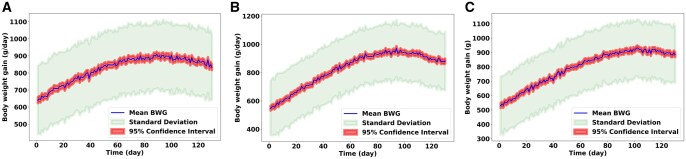
Changes in pigs’ body weight gain (BWG) from 500 replications of the model. The line depicts the mean BWG trajectory for 500 replications. The shaded area represents the standard deviation, illustrating the variability in simulated outcomes due to the model’s stochastic elements. The larger shaded area shows the 95% confidence interval of the mean BWG. (A) Estimated BWG for gilts, (B) for barrows, (C) for boars.

The simulations demonstrated the capabilities of the SNS model to indicate the growth paths of different pigs. The BWG depends on various factors, including sex and age. Figure 3 illustrates the stochastic nature of the SNS model to simulate a pig’s BWG over 130 d. The blue line represents the BWG trajectory calculated across these 500 independent runs, estimating the expected population-level growth. The shaded light green area visualizes the standard deviation around this mean, demonstrating the range of potential BWG outcomes resulting from the model’s stochasticity. Furthermore, the shaded light red area depicts the 95% confidence interval of the mean BWG. This interval provides a measure of the uncertainty associated with the estimated average BWG, indicating the range within which the true mean BWG is likely to happen 95% of the time, given the stochastic nature of the model and the number of simulations performed. The increasing spread of the standard deviation and the confidence interval over time suggests that the cumulative effect of the model’s stochastic elements leads to more significant divergence in individual and average growth trajectories as the simulation progresses, highlighting the importance of considering this variability when interpreting the model’s predictions. This result indicates that the SNS model can mimic the dynamic behavior of real-world swine systems.

**Figure 4.**
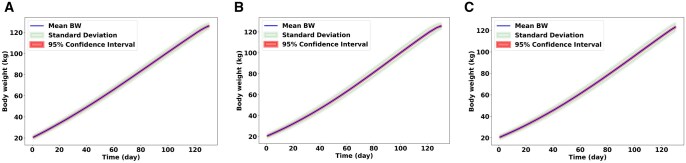
Changes in pigs’ body weight (BW) for 500 replications of the model. The line depicts the mean BW trajectory for 500 replications. The narrow shaded area represents the standard deviation, illustrating the variability in simulated outcomes due to the model’s stochastic elements. The broad shaded area shows the 95% confidence interval of the mean BW. (A) Estimated BW for gilts, (B) for barrows, (C) for boars.

**Figure 5.**
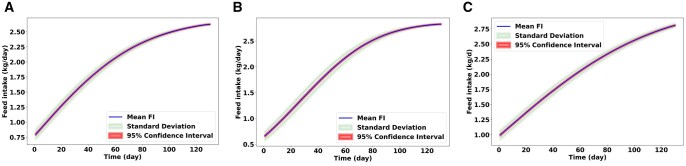
Changes in pigs’feed intake (FI) for 500 replications of the model. The line depicts the mean FI trajectory calculated across 500 replications. The small shaded area represents the standard deviation, illustrating the variability in simulated outcomes due to the model’s stochastic elements. The large shaded area shows the 95% confidence interval of the mean FI. (A) Estimated FI for gilts, (B) for barrows, (C) for boars.

Figure 4 shows the simulated average BW of gilts, barrows, and boars over 130 d based on 500 independent model runs. The blue line indicates the mean BW, reflecting the average growth of the pig population as predicted by the model. The light green shaded area represents the standard deviation around the mean, highlighting the range of potential BW outcomes resulting from the model’s stochastic elements. The light red shaded area also illustrates the 95% confidence interval of the mean BW. The relatively narrow standard deviation and confidence interval throughout the simulation period imply a consistent average growth pattern captured by the model across multiple runs. Figure 5 illustrates the average daily FI of three sexes of pigs over a 130-day simulation period, derived from 500 runs of the model. The standard deviation around the mean FI illustrates the range of deviation in daily feed consumption across the simulations. Similarly, the 95% confidence interval of the mean FI also contracts as the simulation progresses. This indicates that our estimate of the true average daily FI becomes more precise over time, likely due to the stabilization of FI patterns across the simulated population after the initial growth phase. The convergence of these variability measures suggests that while the model incorporates stochasticity, the overall FI behavior of the simulated pig population becomes more predictable and less variable in the later stages of the simulation. Other parameters, such as Pd, Ld, and MEI, have been evaluated as well, which can be found in the Supplementary Figures S1 to S3, respectively.

**Figure 6.**
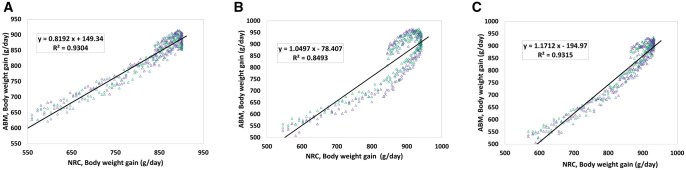
(A) Correlation between body weight gain (BWG) calculated by the National Research Council (NRC) model and BWG predicted by the Swine Nutrition System (SNS) model for gilts, (B) for barrows, (C) for boars. Different colors refer to distinct replications. The solid line and the equation refer to the linear regression fitted to the data (NRC, 2012).

Known differences in the growth performance of barrows, gilts, and boars provide an opportunity for model evaluation between agent attributes. Simulations were repeated for all three sexes to confirm the repeatability for all pigs. Figure 6 presents the correlation between BWG calculated by the NRC and predicted by the proposed SNS for gilts, barrows, and boars. The simulations were repeated three times for each sex with the same conditions, shown by different colors. Of particular interest is the strong positive correlations among all sex classes, as confirmed by each having relatively high coefficient of determination (*R*^2^) values. Hence, this strongly indicates that the model truly represents the interaction of these other factors affecting pig growth, including FI, nutrient absorption, and metabolic activities.

**Figure 7.**
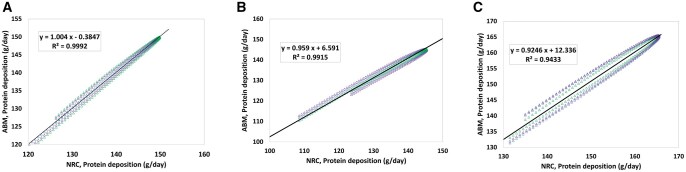
(A) Correlation between protein deposition (Pd) calculated by the National Research Council (NRC) and Pd predicted by the Swine Nutrition System (SNS) model for gilts, (B) for barrows, (C) for boars. Different colors refer to distinct replications. The solid line and the equation refer to the linear regression fitted to the data (NRC, 2012).

Protein is a critical growth factor, being required in various physiological processes in the pig body; thus, Pd has to be taken into account for proper studies of growth and development in pigs. Figure 7 compares Pd computed by the NRC with predicted Pd from the SNS. The accurate reflection of protein metabolism is crucial for determining the maximum dietary protein levels and subsequent lean muscle development. The strong positive correlations among all sexes indicate the model’s accuracy in simulating the processes of protein synthesis and deposition in the growing-finishing pig.

**Figure 8.**
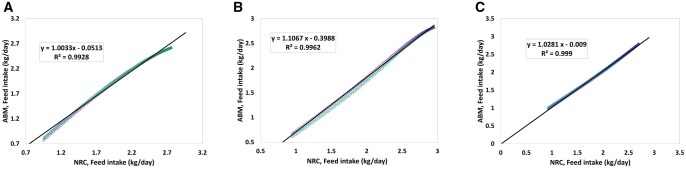
(A) Correlation between feed intake (FI) calculated by the National Research Council (NRC) and FI predicted by the Swine Nutrition System (SNS) model for gilts, (B) for barrows, (C) for boars. Different colors refer to distinct replications. The solid line and the equation refer to the linear regression fitted to the data (NRC, 2012).

The FI values evaluated by the NRC (2012) in comparison with those predicted by the SNS are shown in Fig. 8. Strong positive correlations were observed in all three sexes further validating the model’s ability to predict correctly the FI patterns. Therefore, this representation of FI is an important step toward optimizing feeding strategies and adequate nutrient delivery. The correlation of other attributes, including Ld and MEI, has also been evaluated, which can be found in the Supplementary Figures S4 and S5, respectively.

As SID lysine is a benchmark for formulating balanced diets that meet the specific lysine requirements of pigs at different stages of growth, it is highly crucial in swine nutrition models. SID lysine accounts for the variation in amino acid digestibility along the pig’s digestive tract (De La Llata et al., 2007). Unlike total lysine, which does not consider the differences in amino acid digestibility, SID lysine considers the digestibility of lysine in the small intestine, where most nutrient absorption occurs. Inclusion of SID lysine in swine diets affects the requirement for some other indispensable amino acids. Studies proved the SID lysine effect on the requirement of pigs for vital amino acids (e.g. arginine, histidine, isoleucine, leucine, methionine, phenylalanine, threonine, tryptophan, and valine) (NRC, 2012). Due to the interrelationship of SID lysine with these amino acids, it plays a substantial role in adjusting swine feed nutrition compositions to fulfill growth, development, and general health in pigs. Table 5 compares model-derived estimates of SID amino acid requirement, STTD P, and total Ca for gilts, barrows, and boars fed to day 130.

**Table 5.** Estimations of standardized ileal digestible (SID) amino acid requirements, standardized total tract digestible (STTD) P, and total Ca for gilts, barrows, and boars from the Swine Nutrition System model on day 130 of feeding

	Gilt	Barrow	Boar	
**BW (kg)**	126.55	127.99	123.55	
**SID Lysine (g/d)**	17.65	17.31	19.47	
**SID amino acids (requirements relative to lysine) (g/d)**				**% of SID lysine**
**Arginine**	8.06	7.91	8.90	45.7
**Histidine**	6.06	5.95	6.70	34.4
**Isoleucine**	9.21	9.03	10.16	52.2
**Leucine**	17.76	17.43	19.61	100.7
**Methionine**	5.09	5.00	5.62	28.9
**Methionine + cysteine**	9.95	9.76	10.98	56.4
**Phenylalanine**	10.53	10.33	11.62	59.7
**Phenylalanine + tyrosine**	16.55	16.24	18.27	93.8
**Threonine**	10.64	10.44	11.74	60.3
**Tryptophan**	3.01	2.96	3.33	17.1
**Valine**	11.45	11.23	12.64	64.9
***N***	37.90	37.19	41.83	214.8
**Phosphorus and calcium requirements (g/d)**				
**STTD P**	5.34	5.08	6.22	
**Total Ca**	11.49	10.92	13.38	

Minerals are required for the formation of bones and teeth, the regulation of enzyme systems, the maintenance of acid–base balance, and proper nerve and muscle function (Fadlalla, 2022), and they play a crucial role in the swine nutrient system. Minerals are also involved in hormone production, immune function, and the transportation of oxygen throughout the body, which makes them necessary in the diet to ensure optimal growth performance (Weyh et al., 2022; Sampath et al., 2023). The proposed model estimates the mineral requirements for swine based on the individual attributes and interactions. These incorporate animal age, BW, growth rate, physiological status of the animal under consideration, and environmental factors, including the production system. Hence, the model can correctly estimate the swine’s mineral requirements during the pig life cycle to ensure these animals receive enough essential minerals. Estimates of minerals are compared for gilts, barrows, and boars on day 130 of feeding in Table 6.

**Table 6.** Estimations of mineral requirements for gilts, barrows, and boars from the Swine Nutrition System model on day 130 of feeding

Mineral	Gilt	Barrow	Boar
**Sodium (g/d)**	2.92	2.94	2.90
**Chlorine (g/d)**	2.31	2.32	2.29
**Magnesium (g/d)**	1.15	1.16	1.14
**Potassium (g/d)**	4.75	4.76	4.73
**Copper (mg/d)**	8.46	8.48	8.41
**Iodine (mg/d)**	0.40	0.40	0.40
**Iron (mg/d)**	111.34	111.52	110.96
**Manganese (mg/d)**	5.79	5.82	5.74
**Selenium (mg/d)**	0.41	0.42	0.41
**Zinc (mg/d)**	140.96	141.45	139.91

In addition to minerals, vitamins are involved in metabolism, immune response, reproduction, and growth, also serving as coenzymes and precursors for important molecules in the body (Mainardi et al., 2024; Shastak and Pelletier, 2024). Swine suffer from various diseases and poor performance with low vitamin levels in their diet. The SNS considers feed composition, levels of nutrients, and biological factors in estimating the specific vitamin needs of swine within a given population. Vitamin estimations are compared among gilts, barrows, and boars on the 130th day of feeding in Table 7, which gives good insights to tailor the nutrient management strategies to meet the individual needs of swine at different stages of growth and production.

**Table 7.** Estimations of vitamin requirements for gilts, barrows, and boars from the Swine Nutrition System model on day 130 of feeding

Vitamin	Gilt	Barrow	Boar
**Vitamin A (IU/d)**	3,767.85	3,784.44	3,732.48
**Vitamin D3 (IU/d)**	434.76	436.67	430.68
**Vitamin E (IU/d)**	31.88	32.02	31.58
**Vitamin K (mg/d)**	1.44	1.45	1.43
**Biotin (mg/d)**	0.14	0.14	0.14
**Choline (g/d)**	0.86	0.87	0.86
**Folacin (mg/d)**	0.86	0.87	0.86
**Niacin (mg/d)**	86.95	87.33	86.13
**Pantothenic acid (mg/d)**	19.89	19.96	19.73
**Riboflavin (mg/d)**	5.59	5.61	5.56
**Thiamin (mg/d)**	2.89	2.91	2.87
**Vitamin B6 (mg/d)**	2.89	2.91	2.87
**Vitamin B12 (μg/d)**	12.51	12.50	12.53
**Linoleic acid (g/d)**	2.89	2.91	2.87

**Figure 9.**
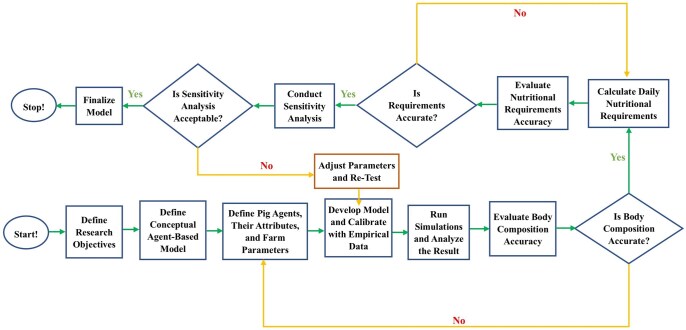
A flowchart illustrating the sequential steps involved in developing, calibrating, and evaluating the Swine Nutrition System model.

Transparent and reproducible methodologies are essential for model-based research. Figure 9 shows our modeling protocol, from highlighting primary decision points and iteration processes to specific evaluations to ensure the accuracy and validity of the model. This flowchart depicts how the presented SNS was developed, calibrated, and evaluated. This protocol started with the definition of the research objectives and the conceptualization of the ABM framework. Thereafter, we defined the attributes, behaviors, and interaction characteristics of the pig agents and relevant farm (e.g. environment) parameters. Subsequently, the SNS model was executed under a series of pre-set simulations, post-processed, and analyzed to determine whether the model effectively predicted pig body composition. If the model results were deemed acceptable, the daily nutritional requirements of pigs were calculated and verified for compliance with previously established nutritional standards. Finally, sensitivity analyses were conducted to identify parameters with the most significant influence or effect on model output. An iterative procedure was followed to allow for continual model reinterpretation, parameterization, and re-testing. By subjecting the output to an iteration of the steps described here, we establish that the resultant SNS constitutes a start in good faith toward modeling the system of swine nutrition. The findings showed that selecting an ABM framework for our SNS provided a new ability to capture individual animal differences and the changing dynamics over time, including pig behavior and their interactions with the environment. This has been a challenge for traditional models that aggregate data. Deterministic approaches like the NRC (2012) can give accurate population-level predictions, but they lack the ability to incorporate biological variability or to simulate emergent behaviors arising from individual differences in FI, metabolism, and social interactions. Using ABM, we could include features such as sex-specific parameters and the effects of environmental stressors like temperature, which influence energy needs and growth. Although ventilation was not explicitly modeled due to the study’s scope, the modular design of the SNS allows for future addition of more factors. Employing an ABM approach is therefore a scalable way to develop precision nutrition strategies that balance biological realism and computational efficiency, producing individualized feeding options that support economically sustainable production without relying on costly experimental trials.

## Implications and Applications

The SNS model developed using an ABM framework in this study represents an important advancement in pig nutrition and growth modeling because it enables the simulation of individual animal behavior, nutrient partitioning, and variability in performance. These capabilities are not possible with traditional aggregate models. By leveraging ABM, the SNS can capture the complex, dynamic interactions between pigs and their environments, including management practices and environmental stressors, offering a powerful, flexible platform for exploring innovative strategies in swine production.

One of the strengths of the proposed SNS is that it allows the simulation of pig behavior and dynamic nutrient partitioning at the individual level. By representing pigs as autonomous agents with their own unique characteristics and responses to the environment, the model can capture the variability in growth trajectories and nutrient utilization that is often overlooked in traditional approaches. This level of detail may be particularly valuable for swine industry decision-makers, as the model has the power to provide insight into underlying mechanisms driving nutrient use and utilization in differing production settings.

In addition to simulating individual pig behavior, the model also enables the assessment of various environmental stressors (e.g. ambient temperature outside of the pig’s thermoneutral zone) and management strategies. This is particularly relevant in modern swine production, in which factors such as housing conditions and feeding strategies impact a farm’s efficiency, profitability, and sustainability. By incorporating these factors into the model, researchers and industry professionals may easily and rapidly simulate multiple scenarios to identify the largest costs and opportunities. Indeed, the virtual nature of the SNS provides a risk-free and expense-free environment for producers, scientists, nutritionists, consultants, and students to test multiple scenarios and interventions. For example, the pig growth performance and nutrient requirement results reported herein were derived from a simulation of pigs growing from 20 to 130 kg. These default settings are appropriate for model demonstration but would fail to represent the variation of natural conditions. It is well known that animal growth and farm profit are influenced by pig-specific factors, such as birth weight and weaning weight (Cabrera et al., 2010; Fix et al., 2010), and marketing decisions such as sorting at pig removal to market (Cheng et al., 2017; Zhou and Bohrer, 2019). The capability of the SNS to simulate individual pigs (for example, to test the impact of increasing standard deviation of pig BW at barn stocking on farm nutrient use) and user customizations (for example, to evaluate the effect of different strategies to remove pigs from a barn at marketing) make this program the ideal experimental software to allow for exploration and evaluation of innovative ideas and management practices without incurring the costs and risks associated with real-world experimentation. By providing a platform for *in silico* experiments, the model may even accelerate the pace of innovation in pig production while facilitating rapid evaluation of new ideas and technologies that drive continuous improvement.

No model is ever complete, but it must be continuously improved to reflect updated conditions. The ABM was built with a modular design to enhance the utility and adaptability. In fact, by breaking down the model into discrete components that can be easily modified or expanded, the model managers may regularly and easily update the material with new empirical data, revised mechanistic equations, or additional modules to address user needs or questions. This flexibility helps to ensure that the model will remain relevant and up-to-date in a rapidly evolving field. Future developments of the model will enhance its value to swine producers. Primarily, the model should integrate with existing databases or models. Integration with pig performance data could allow for better refinement of the stochastic elements of the model and a truer representation of modern pig farms. Additionally, integration with existing feed optimization models would allow for exploration of feeding strategies and better quantification of sustainability metrics.

In the version outlined in this report, the model assumes that the pig’s nutrient requirements are perfectly met on each day; in reality, that would not be feasible. Instead, pigs are commonly fed in phases with diets formulated to meet the average requirement of the population. This strategy is effective but less efficient than the results represented herein (Pomar et al., 2014). Accordingly, user inputs of diet specifications would allow the incorporation of several important considerations, such as feed ingredient digestibility, instances and severity of under- and over-feeding, and nutrient excretion in manure. Further, when nutrient parameters are defined by a user, nutrient flow through a system may be quantified. This would similarly allow the assessment of economic performance alongside the environmental impacts. By integrating life cycle assessments and feed optimization models within the system, we can complete multiple evaluations to identify the most cost-effective strategies that minimize ecological impacts.

## Conclusions

This study represents an advancement in swine production modeling by successfully developing and implementing an ABM capable of simulating individual pig growth, FI, metabolism, and nutrient requirements under various environmental conditions and management strategies. By focusing on the growing-finishing phase, the model was verified and found to replicate the NRC (2012) outputs accurately. By leveraging the flexibility and detail offered by ABM, the SNS model provides a dynamic and robust platform for researchers, nutritionists, and producers to evaluate feeding strategies, management practices, and technological interventions in a virtual environment. Beyond its technical contributions, the SNS model aligns with broader industry priorities of resource stewardship, environmental conservation, and long-term sustainability. As such, it offers a valuable framework for supporting informed decision-making, promoting continuous improvement, and developing innovative solutions to the complex challenges facing modern pig production systems.

## Supplementary Material

skaf443_Supplementary_Data

## Data Availability

The agent-based model developed for simulating swine feeding and growth performance, along with its interactive interface, is publicly available at https://animalnutrition.org/node/14271. This repository includes the source code, documentation, and user guide to support replication and further research. All data and software used in this study are accessible and comply with the Journal of Animal Science’s data sharing policies.

## Abbreviations:

ABM: agent-based modeling
BA: whole-body ash mass
BL: whole-body lipid mass
BP: whole-body protein mass
BW: body weight
BWG: body weight gain
BWt: whole-body water
Ca: calcium
FDM: feed dry matter
FDMI: feed dry matter intake
FI: feed intake
GIT: gastrointestinal tract
Gutf: gut fill
IoT: Internet of Things
LCT: lower critical temperature
Ld: lipid deposition
ME: metabolizable energy
MEI: metabolizable energy intake
NRC: National Research Council
P: phosphorus
PBT: probe backfat thickness
Pd: protein deposition
SID: standardized ileal digestible
SNS: swine nutrition system
STTD: standardized total tract digestible
