# Supplementary material for: ASAS-NANP symposium: mathematical modeling in animal nutrition: agent‑based modeling of nutrient requirements and growth performance in growing–finishing pigs for sustainable production systems
Source: J Anim Sci. 2025 Dec 18;104:skaf443. doi: 10.1093/jas/skaf443 (PMC12924631; doi:10.1093/jas/skaf443)
Supplement: skaf443_Supplementary_Data [file skaf443_supplementary_data.zip › Supplemental_Material-Revised.docx]

**Supplemental Material**

Figure 1 includes the average estimated Pd for gilts, barrows, and boars across a 130-day simulation. The blue line represents the average Pd, which peaks at approximately the middle of the growing time and then declines steadily to the end of the simulation. The light green shaded area surrounding the mean Pd represents one standard deviation, which was narrow throughout most of the simulation, indicative of consistency with low variation across the 500 replicates. The light red shaded area also represents the 95% confidence interval of the mean Pd, which provides additional confidence that the estimates provided by the SNS are stable and reliable. Figure 2 presents the estimated average Ld for three sexes of pigs over a 120-day simulation period. The blue line represents the mean Ld, which shows a clear upward trend. This indicates effective nutritional growth as the pigs mature. The light green shaded area around the mean line illustrates the standard deviation, which narrows as the pigs mature, suggesting more consistent predictions with lower variation. Additionally, the 95% confidence interval of the mean Ld reflects high confidence in these estimates and stability throughout the proposed model. The estimated MEI for gilts, barrows, and boars for 500 repetitions is shown in Fig. 3. The mean MEI increases steadily, which reflects increasing energy requirements associated with the pig's maturity. Figure 3 also demonstrates a small amount of variation around the mean MEI throughout the lifespan of the pigs, which shows that there was small variability across the 500 replications.

To statistically evaluate and examine the repeatability of the proposed model for other important parameters of the pigs, we simultaneously simulated three pigs of each sex with the same age and conditions and analyzed their fundamental attributes such as parameters such as Ld and MEI. Simulations were repeated for all three sexes to confirm the repeatability for all pigs, which were shown by different colors. The statistical evaluations are shown in Figure 4 and Figure 5, respectively. In the same way, Fig. 4 shows the correlation between Ld calculated by the NRC and predicted by the ABM. The consistently strong positive correlations across all sexes demonstrate the capacity of the model to accurately simulate lipid deposition patterns. This is particularly significant as lipid deposition varies considerably among different pigs concerning the sex and genotype, resulting in significant implications for carcass quality and overall economic performance. The correlations between MEI intake calculated by the NRC and that predicted by the ABM for all three sexes have been explored in Fig. 5. Strong positive correlations appear in this study to suggest that the model successfully reproduces the energy requirements of growing-finishing pigs. This results in an accurate representation of energy utilization important in minimizing energy wastage in formulation.

The simulations demonstrated the capabilities of the SNS model to indicate the growth paths of different sexes of pigs. In Fig. 6 (a), the graphs depict the progression of BWG for each sex, and showcase the unique trends in BWG across time. The model results have represented the recognized variations in growth rates and body structure among the sexes. In order to well understand the capability of the proposed model in the individual pigs’ BW, Fig. 6 (b) shows the BW of growing pigs for three different sexes. This figure demonstrates a steady increase in BW as the pigs mature, with some day-to-day fluctuations due to their active movement and consistent feeding which looks at the actual BWG over time, complementing the broader growth trends. By examining the detailed BW changes on a daily basis, the model can capture the nuances of the pigs’ growth patterns, accounting for factors such as their activity levels and FI. A comparative analysis of Pd as trends over time among gilts, barrows, and boars is in Fig. 6 (c). The three groups differed remarkably in Pd profiles. The boars depict the highest Pd of the three, which means that, on the whole, they have a greater Pd as compared to the other two sexes. The gilts curve the Pd moderately, while throughout growth, the lowest Pd is seen in the barrows. Pd characteristics mirror the physiological and hormonal variations that regulate protein metabolism in pigs of different sexes and therefore muscle development. Such an understanding of Pd variance becomes most important in feeding strategy design and nutrition programs to meet the growth and carcass composition goals targeted for each sex of pigs. Assessment of increasing Ld trends presented in Fig. 6 (d) reveals a good distinction in growth for gilts, barrows, and boars with respect to age in pigs. The Ld curves simulated to show that the female pigs (barrows and gilts) had a much quicker increase in Ld, reaching peaks of approximately 409 g/day and 359 g/day respectively, at 130 days of age. Contrary to that, the Ld of boars is still increasing but at a slower pace with peaks less than others, around 314 gm/day at that same age. All these signals some variety in the physiological differences reflected in growth patterns for different sexes of pigs. Such differentials in Ld curves give insight highlighting how principles on growth patterns based on sex must be used in developing feeding strategies and management schemes to grow pigs rapidly and efficiently.

Fig. 7 (a) shows the changes in MEI for the three sexes of pigs. It can be seen that while pig grew up the amount of MEI increases for all three types of pigs. However, the amount of MEI for barrows is the highest around all the time and the lowest for boars. Another point to mention is that at young ages, the increase in MEI occurs with a rapid slope and, after the pig has attained roughly half of its ultimate live BW, the slope decreases gradually. A comparison of maintenance ME requirements for whole sexes along the farm has been considered during their lifetime in Fig. 7 (b). Assessing and estimating such parameters would guide producers and nutritionists toward accuracy in determining energies for maintenance, with the balance directed toward production, growth, or other physiological functions.

Figure 8 dynamically illustrates the changes in FI for gilts, barrows, and boars. As one can see, changes in FI are completely different for all the pigs. For instance, males consume more than other sexes during the first days of feeding. Their FI increases less than the FI of other sexes in the middle ages. Meanwhile, in the older age group, the slope of FI increases. It shows that producing boars is more expensive.

The results of changing this vital parameter during the animal lifespan have been illustrated in Fig. 9. The figure clearly shows higher requirements of SID lysine for boars in contrast with gilts and barrows. Regarding the cost of amino acids, it proves how expensive raising boars is in comparison with gilts and barrows as pork production. By evaluating SID lysine requirements, nutritionists can improve feed efficiency, minimize nitrogen excretion, and promote sustainable pork production. By feeding rations based on SID lysine requirements, producers can minimize costs while maximizing performance.

Table 1 represents the coefficients *p* and *q* for minerals and *m* and n for vitamins applied in equations (3-10) and (3-11) to calculate mineral and vitamin requirements, respectively, for each pig considering their weight.


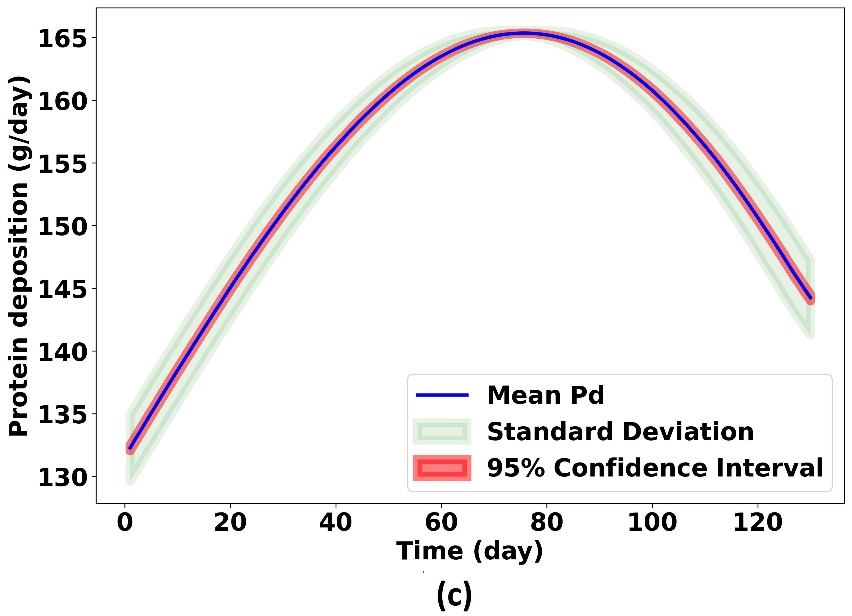

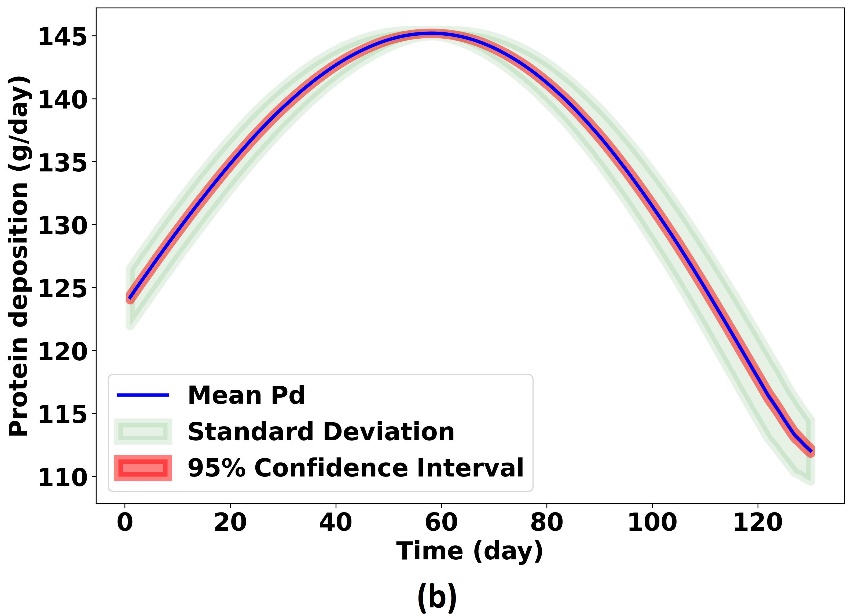
**Figure 1** Changes in pigs’ Pd during the simulation time for 500 replications. The blue line depicts the mean Pd trajectory calculated across 500 replications. The light green shaded area represents the standard deviation, illustrating the variability in simulated outcomes due to the model’s stochastic elements. The light red shaded area shows the 95% confidence interval of the mean Pd. (a) Estimated Pd for gilts. (b) Estimated Pd for barrows. (c) Estimated Pd for boars.
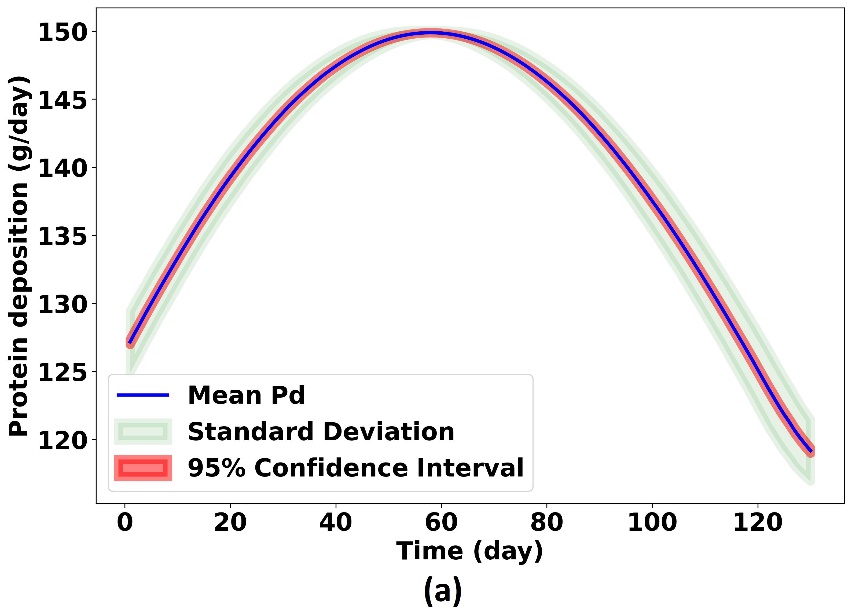


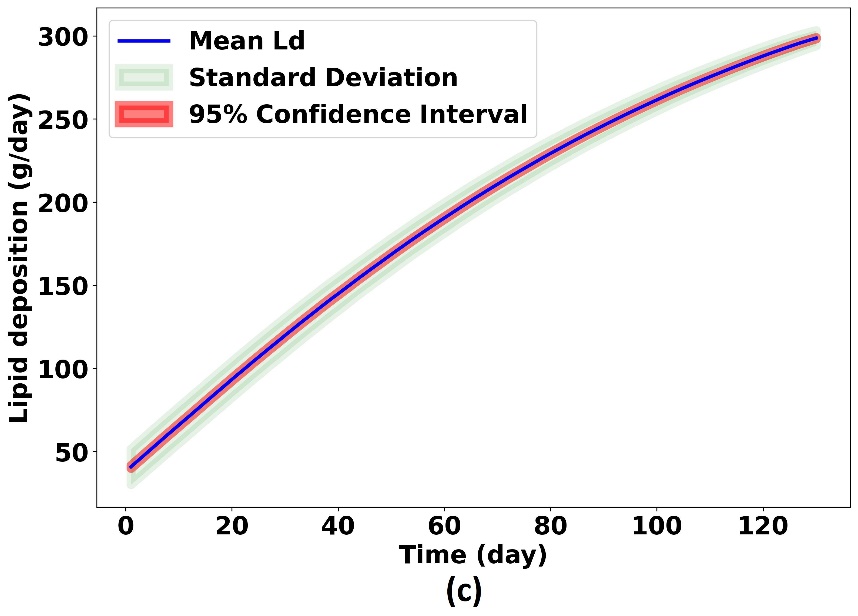

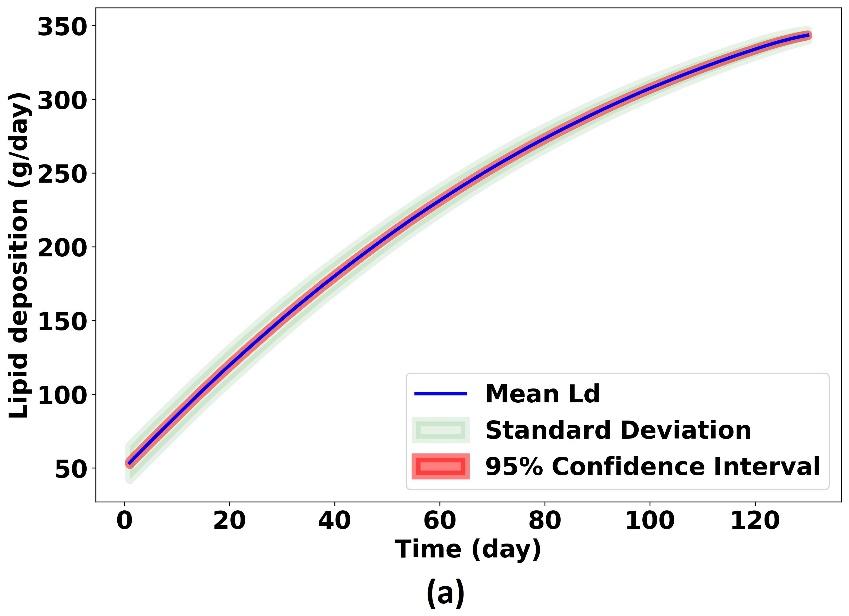

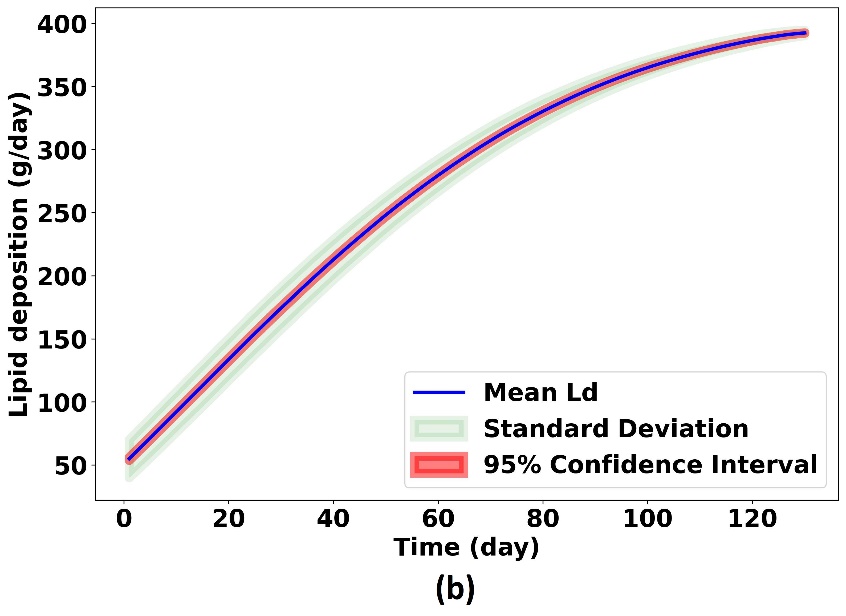
**Figure 2** Changes in pigs’ Ld during the simulation time for 500 replications. The blue line depicts the mean Ld trajectory calculated across 500 replications. The light green shaded area represents the standard deviation, illustrating the variability in simulated outcomes due to the model’s stochastic elements. The light red shaded area shows the 95% confidence interval of the mean Ld. (a) Estimated Ld for gilts. (b) Estimated Ld for barrows. (c) Estimated Ld for boars.


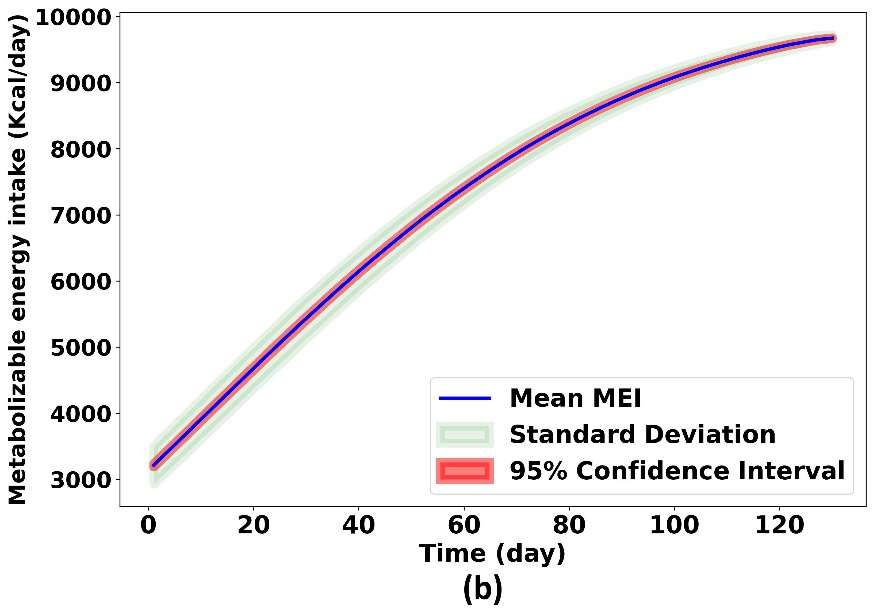

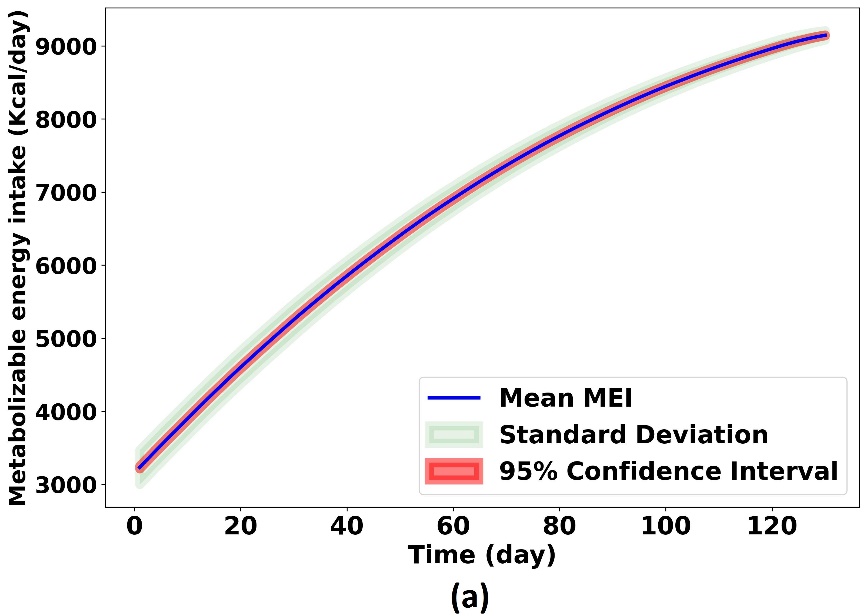

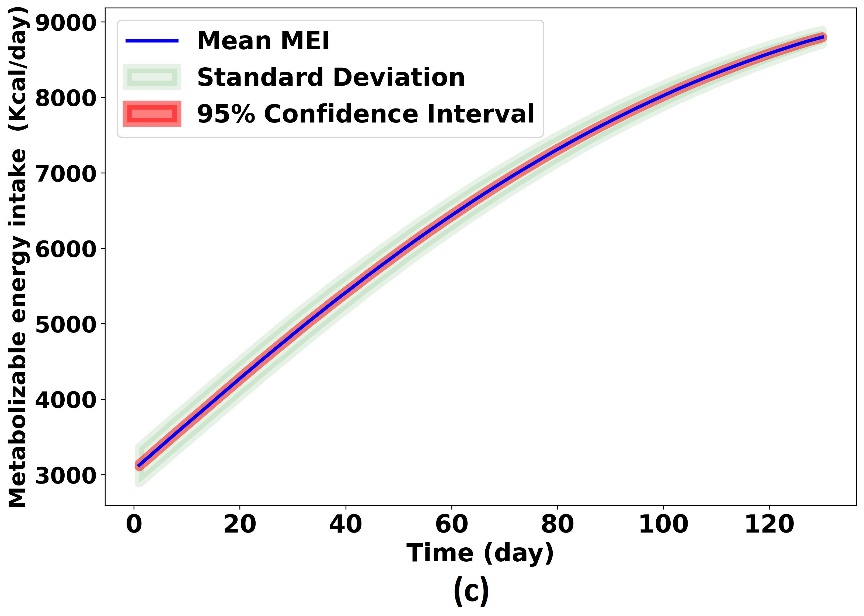
**Figure 3** Changes in pigs’ MEI during the simulation time for 500 replications. The blue line depicts the mean MEI trajectory calculated across 500 replications. The light green shaded area represents the standard deviation, illustrating the variability in simulated outcomes due to the model’s stochastic elements. The light red shaded area shows the 95% confidence interval of the mean MEI. (a) Estimated MEI for gilts. (b) Estimated MEI for barrows. (c) Estimated MEI for boars.


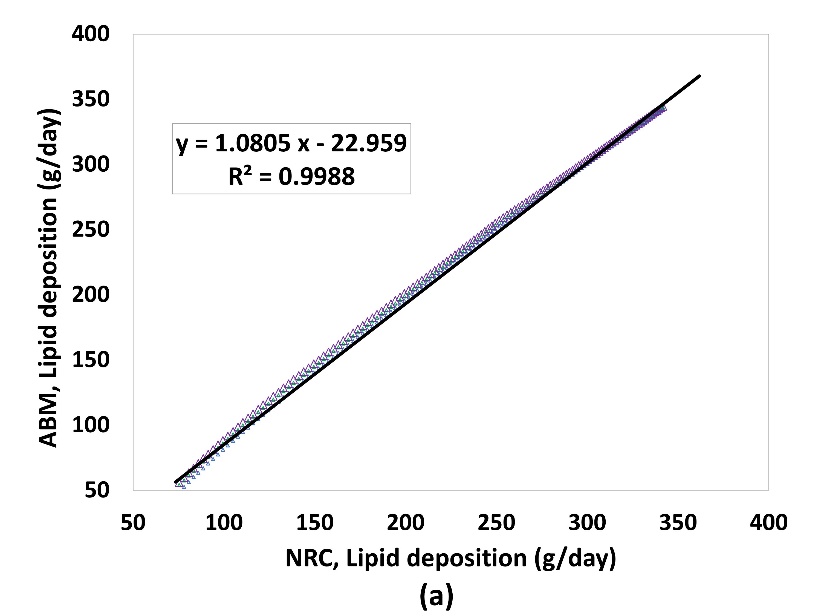

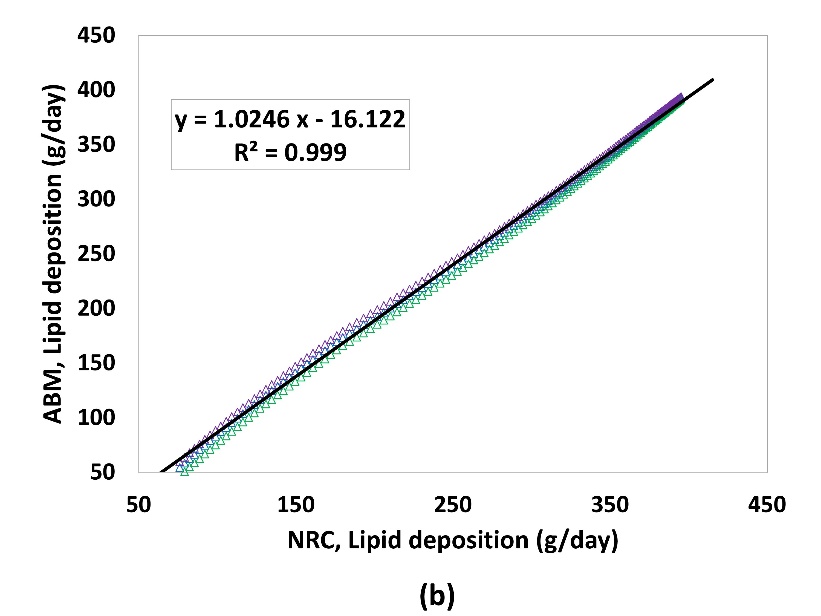

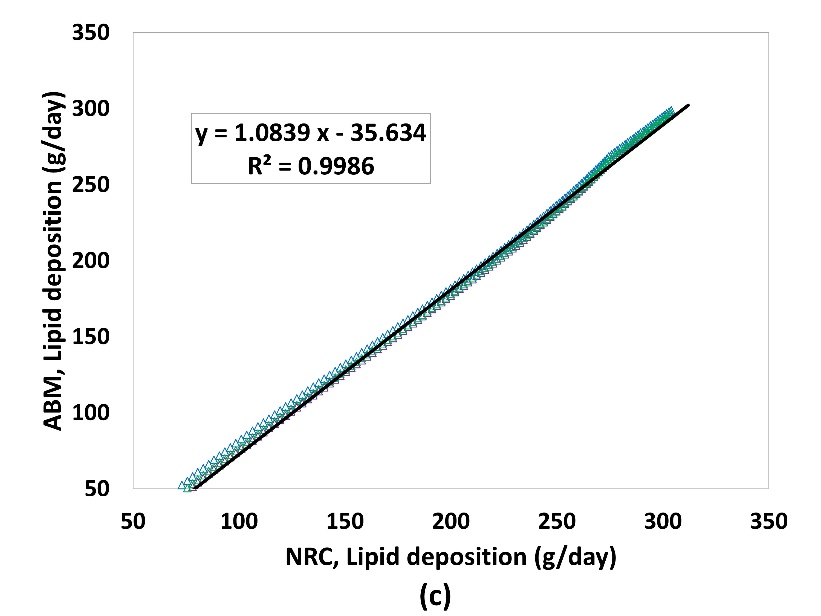
**Figure 4** (a) Correlation between Ld calculated by NRC and Ld predicted by the proposed ABM for gilts. (b) Correlation between Ld calculated by NRC and Ld predicted by the proposed ABM for barrows. (c) Correlation between Ld calculated by NRC and Ld predicted by the proposed ABM for boars. Different colors refer to distinct repetitions. Solid line and its equation refer the linear regression fitted to the data (NRC, 2012).


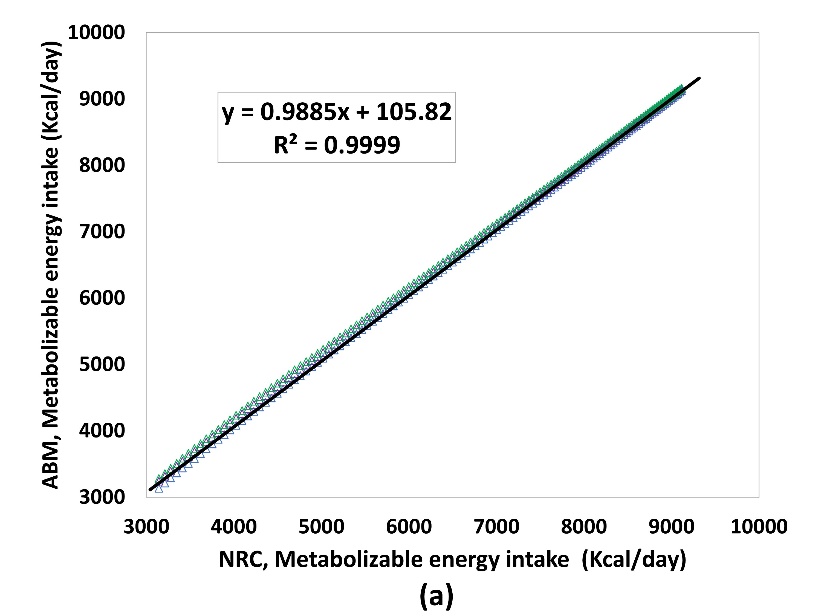

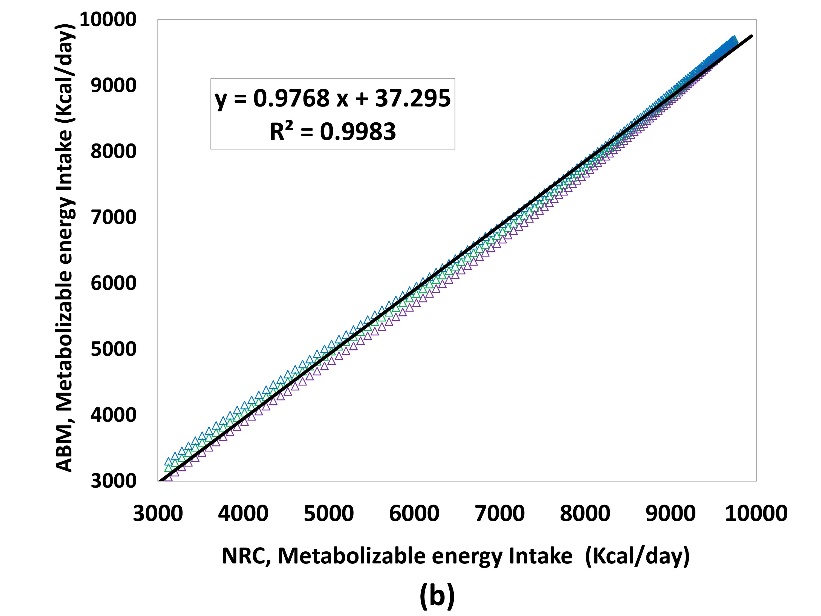

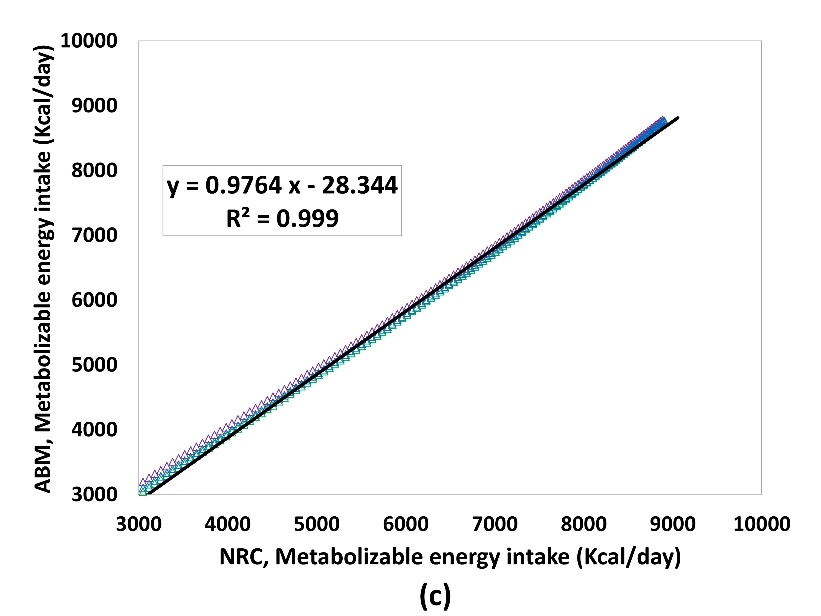
**Figure 5** (a) Correlation between ME intake calculated by NRC and ME intake predicted by the proposed ABM for gilts. (b) Correlation between ME intake calculated by NRC and ME intake predicted by the proposed ABM for barrows. (c) Correlation between ME intake calculated by NRC and ME intake predicted by the proposed ABM for boars. Different colors refer to distinct repetitions. Solid line and its equation refer the linear regression fitted to the data (NRC, 2012).


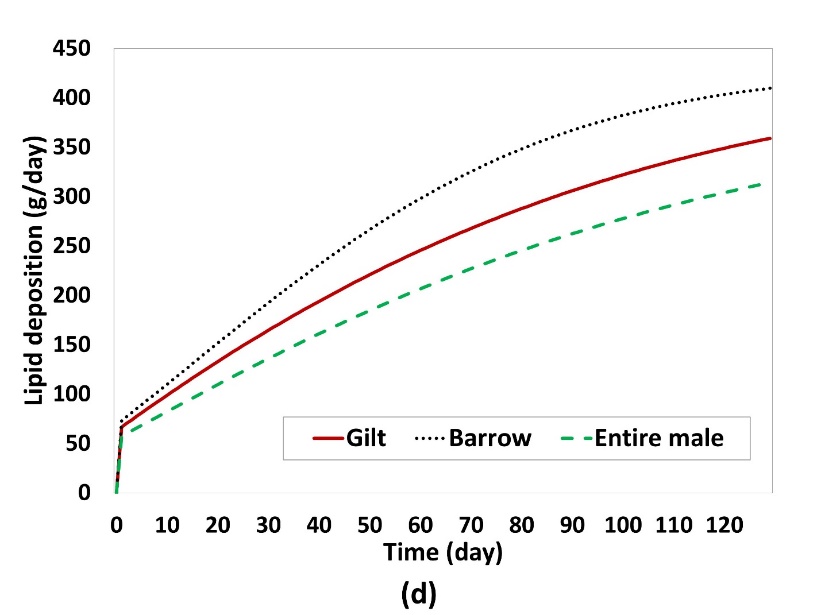

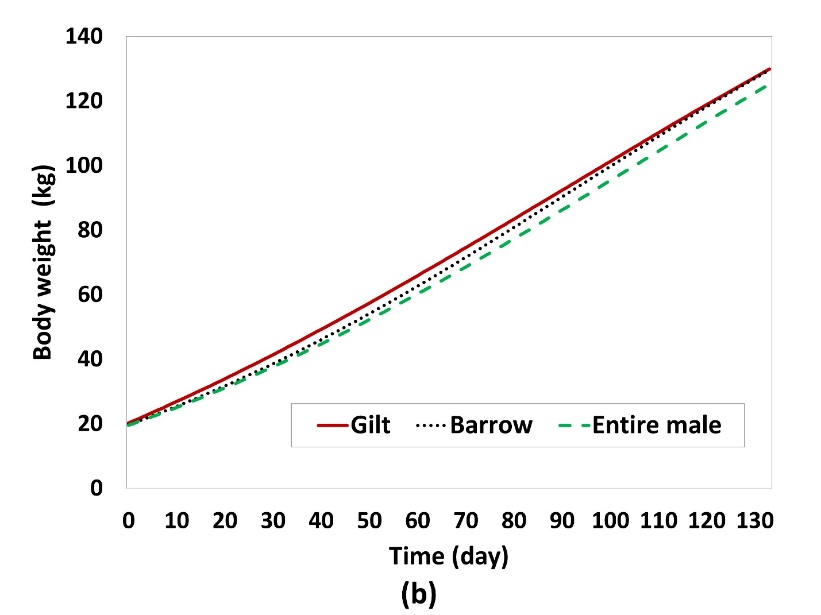

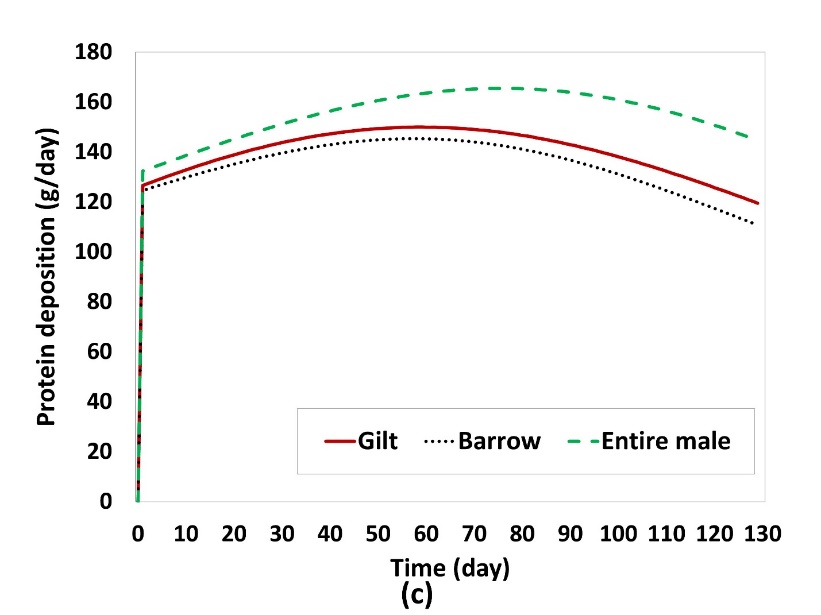

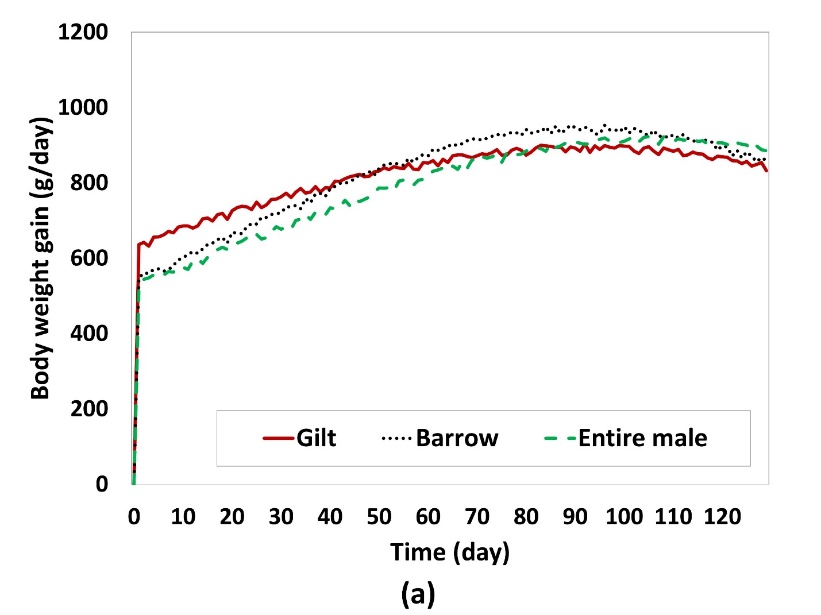
**Figure 6** Comparison of body composition performance curves for individual pigs including gilts, barrows, and boars (a) BWG, (b) BW, (c) Pd, and (d) Ld.


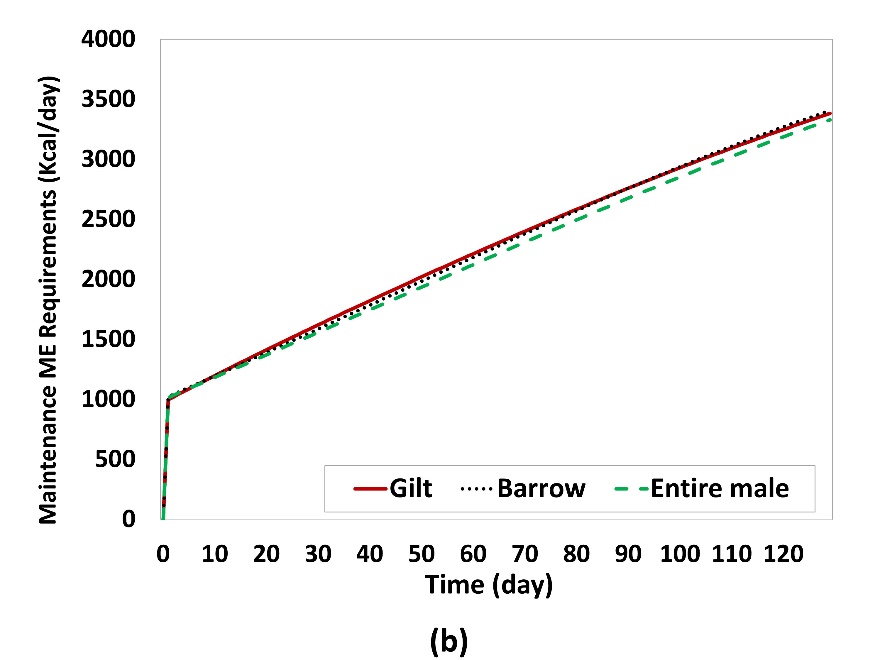

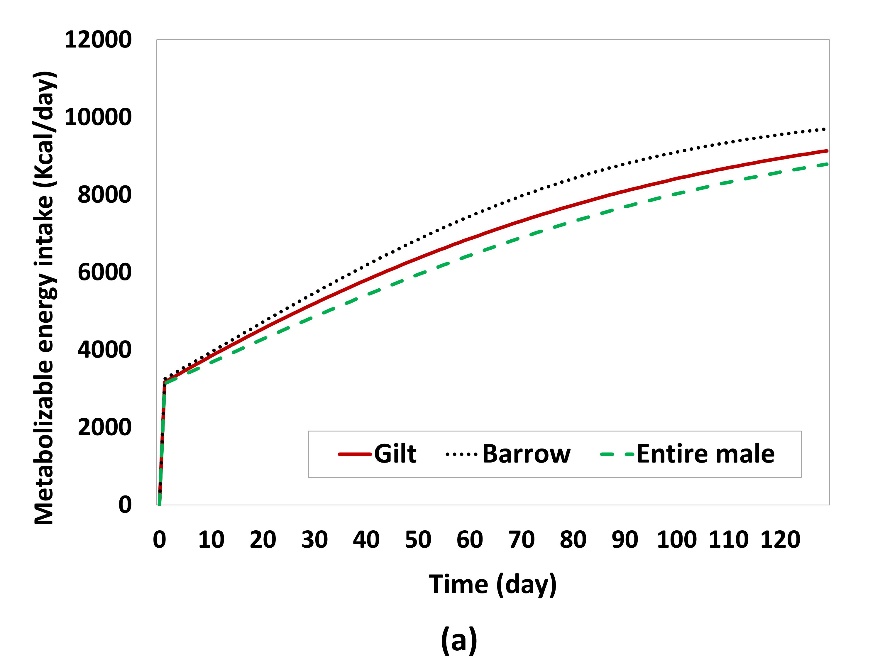
**Figure 7** Comparison of energy intake curves for individual pigs including gilts, barrows, and boars (a) MEI, (b) maintenance ME requirements.

**Figure 8** Average daily FI for gilts, barrows, and boars.


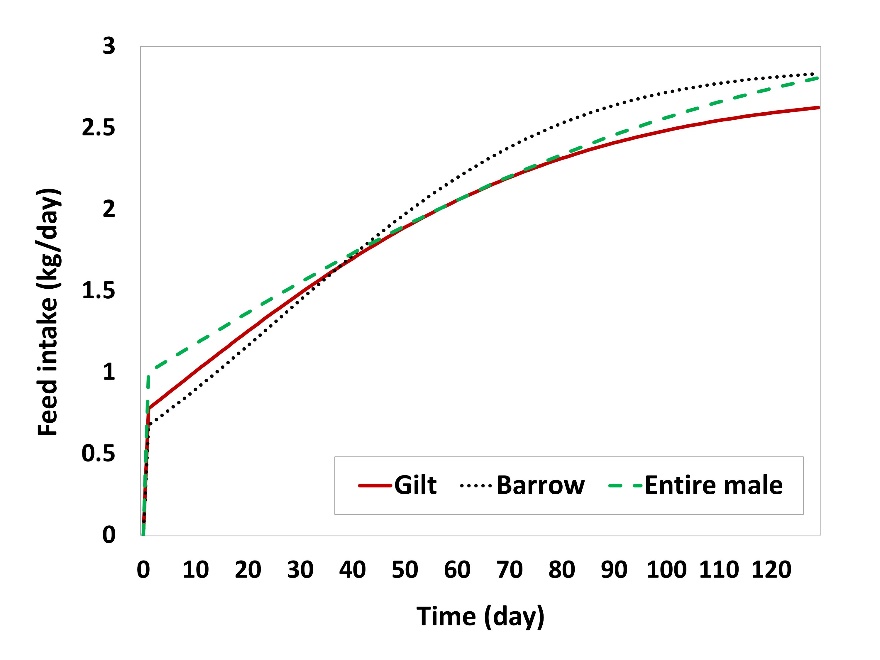


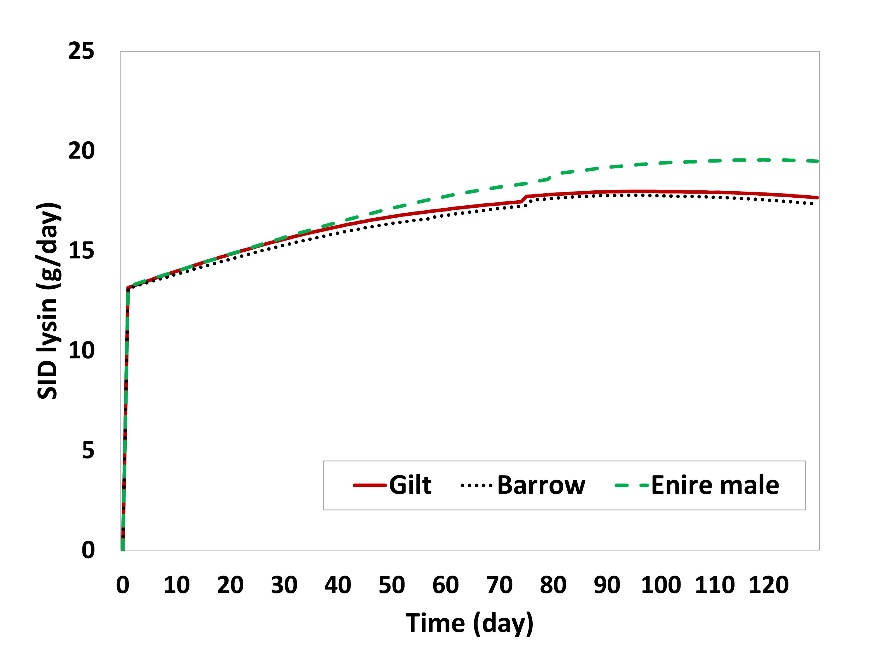
**Figure 9** Comparison of SID lysin for gilts, barrows, and boars.

**Table 1:** Constant coefficients for calculating nutrient requirements of growing finishing pigs

| **Nutrient** | | | | **Coefficient** | | | | |
| --- | --- | --- | --- | --- | --- | --- | --- | --- |
| **Mineral (*x*)** | **Unit** | | ***p*** | | | ***q*** | |  |
| Sodium | | g/day | | | -2.55883 | | 1.1335 | |
| Chloride | | g/day | | | -2.0706 | | 0.9068 | |
| Magnesium | | g/day | | | -1.0353 | | 0.4534 | |
| Potassium | | g/day | | | -0.4591 | | 1.0774 | |
| Copper | | mg/day | | | -0.8705 | | 1.9286 | |
| Iodine | | mg/day | | | -0.3624 | | 0.1587 | |
| Iron | | mg/day | | | 34.357 | | 15.904 | |
| Manganese | | mg/day | | | -5.1766 | | 2.2669 | |
| Selenium | | mg/day | | | -0.092425 | | 0.10483 | |
| Zinc | | mg/day | | | -70.251 | | 43.634 | |
|  | |  | | |  |  |  |  |
| **Vitamin (*x*)** | | **Unit** | | | ***m*** | | ***n*** | |
| Vitamin A | | IU/day | | | -3364.8 | | 1473.5 | |
| Vitamin D | | IU/day | | | -388.24 | | 170.02 | |
| Vitamin E | | IU/day | | | -28.471 | | 12.468 | |
| Vitamin K | | mg/day | | | -1.2941 | | 0.5667 | |
| Biotin | | mg/day | | | -0.1294 | | 0.0567 | |
| Choline | | g/day | | | -0.7765 | | 0.34 | |
| Folacin | | mg/day | | | -0.7765 | | 0.34 | |
| Niacin, available | | mg/day | | | -77.649 | | 34.004 | |
| Pantothenic acid | | mg/day | | | -12.202 | | 6.6304 | |
| Riboflavin | | mg/day | | | -2.2184 | | 1.615 | |
| Thiamin | | mg/day | | | -2.5883 | | 1.1335 | |
| Vitamin B6 | | mg/day | | | -2.5883 | | 1.1335 | |
| Vitamin B12 | | μg/day | | | 16.64 | | -0.852 | |
| Linoleic acid | | g/day | | | -2.5883 | | 1.1335 | |
