# Supplementary material for: ASAS-NANP symposium: mathematical modeling in animal nutrition: agent‑based modeling of nutrient requirements and growth performance in growing–finishing pigs for sustainable production systems
Source: J Anim Sci. 2025 Dec 18;104:skaf443. doi: 10.1093/jas/skaf443 (PMC12924631; doi:10.1093/jas/skaf443)
Supplement: skaf443_Supplementary_Data [file skaf443_supplementary_data.zip › Supplemental_Material_2.docx]

**Appendix 1** A pseudocode that represents the main simulation procedure through the farm

| **Start Procedure:**  **Initialize the simulation**  Set time steps  Set up the simulation environment  Set global parameters  Define necessary parameters  f**or** **all** *time steps* **do:**  **for** **all** *pigs* **do:**  Gather pig’s information  Evaluate the pig’s status (e.g., BW, Pd, Ld, etc.)  Monitor the environmental conditions (e.g., temperature, housing)  Determine the pig’s actions  Move pig through barn  Interact pig with the environment (e.g., consume feed)  Update pig’s internal state based on the actions taken and its individual parameters  i**f** *BW >= final BW****:***  Remove the pig from the farm  Update herd count  **end if**  **end for**  Update the environmental conditions if needed (e.g., temperature, humidity)  Record the simulation outputs (e.g., BWG, body depositions, body requirements)  **end for**  Generate reports and visualizations as needed  **End** |
| --- |

**Appendix 2** A pseudocode illustrating the feeding process and calculations for all pigs

| **// Called from Appendix 1**  **Start Feeding Procedure:**  Set parameters  **for** *all pig categories (gilts, barrows, boars)* **do:**  **for** all pigs in the category **do:**  **if** *gilt***:**  Call ***Feed-g*** (feeding procedure for gilts)  **else if** *barrow*:  Call ***Feed-b*** (feeding procedure for barrows)  **else:**  Call ***Feed-m*** (feeding procedure for boars)  **end if**  **// Common steps for all pig categories**  Calculate pig’s BWG  Update pig’s BW  Calculate pig’s body Composition (e.g. Pd, Ld, BA, BWt, etc.)  Update pig’s energy intake and FI  Update nutrient requirements (e.g. amino acids, minerals, and vitamins)  **if** *feeding ractopamine***:**  Call **Feed-Rac**  **end if**  **end for**  **end for**  **End** |
| --- |
